# Comparative Analysis of Medical Interventions to Alleviate Endometriosis-Related Pain: A Systematic Review and Network Meta-Analysis

**DOI:** 10.3390/jcm13226932

**Published:** 2024-11-18

**Authors:** Ádám Csirzó, Dénes Péter Kovács, Anett Szabó, Bence Szabó, Árpád Jankó, Péter Hegyi, Péter Nyirády, Nándor Ács, Sándor Valent

**Affiliations:** 1 Centre for Translational Medicine, Semmelweis University, 1085 Budapest, Hungary; adamcsirzo@gmail.com (Á.C.); kovacsdenespeter@gmail.com (D.P.K.); a.szabo1995@gmail.com (A.S.); bencetra@gmail.com (B.S.); jankoarpi@gmail.com (Á.J.); hegyi2009@gmail.com (P.H.); nyiradyp@gmail.com (P.N.); acsnandor@gmail.com (N.Á.); 2 Department of Obstetrics and Gynecology, Semmelweis University, 1082 Budapest, Hungary; 3 Department of Urology, Semmelweis University, 1082 Budapest, Hungary; 4 Institute of Pancreatic Diseases, Semmelweis University, 1083 Budapest, Hungary; 5 Institute for Translational Medicine, Medical School, University of Pécs, 7624 Pécs, Hungary

**Keywords:** SERM, NSAIDs, opioids, LNG-IUDs, danazole, VAS score, Biberoglu and Behrman scale, SUCRA

## Abstract

**Background/Objectives**: Endometriosis is a chronic condition that affects 6–10% of women of reproductive age, with pain and infertility being its primary symptoms. The most common aspects of pain are overall pelvic pain, dysmenorrhea, and dyspareunia. Our aim was to compare the available medical treatments for endometriosis-related pain. **Methods**: A systematic search was conducted in three medical databases to assess available drug options for pain management. Randomized controlled trials (RCTs) investigating various medical treatments for endometriosis-related pain on different pain scales were included. Results were presented as p-scores and, in cases of placebo controls, as mean differences (MD) with 95% confidence intervals (CI). From the available data, a network meta-analysis was carried out. **Results**: The search yielded 1314 records, of which 45 were eligible for data extraction. Eight networks were created, and a total of 16 treatments were analyzed. The highest p-score, meaning greatest pain relief (p-score: 0.618), for the treatment of dysmenorrhea was achieved using gonadotropin-releasing hormone (GnRH) agonists for 3 months on a scale of 0–100. Additionally, a p-score of 0.649 was attained following a 6-month treatment with GnRH agonists combined with hormonal contraceptives (CHCs). In the case of dyspareunia on a scale of 0–100 following 3 months of treatment, CHCs (p-score: 0.805) were the most effective, and CHCs combined with aromatase inhibitors (p-score: 0.677) were the best treatment option following 6 months of treatment. In the case of overall pelvic pain, CHCs (p-score: 0.751) yielded the highest p-score on a scale of 0–100 following 3 months of treatment, and progestins combined with aromatase inhibitors (p-score: 0.873) following 6 months of treatment. Progestins (p-score: 0.901) were most effective in cases of overall pelvic pain on a scale of 0–3 following 3 months of treatment. **Conclusions**: Our network meta-analysis showed that in cases of dysmenorrhea, GnRH agonists supplemented with CHCs reduced pain the most following 3 months of treatment. Regarding dyspareunia CHCs were most effective, and in the case of overall pelvic pain, CHCs or progestins combined with aromatase inhibitors yielded the most desirable results.

## 1. Introduction

Endometriosis is a chronic inflammatory disease that depends on estrogen and is marked by the presence of endometrial-like tissue growing outside the uterine cavity [1]. Approximately 10–15% of women of reproductive age groups are affected. The diagnosis is often made only years after the onset of the symptoms, as they are often not specific enough or the only presentation of the disease is infertility [2]. The leading symptoms include chronic pelvic pain, dysmenorrhea, dyspareunia, dyschezia, and dysuria.

Endometriosis-associated pain can be permanent, despite treatment. The cause of this chronic pain is multifactorial, including inflammatory response, nociception, and alterations in pain processing in the peripheral and central nervous systems [3,4]. The psychological aspect of living with chronic pain might be a new perspective in treatment.

Laparoscopy is often the first option both for diagnostic and treatment purposes, enabling the identification of tissues to be removed [5]. However, new approaches are emerging in the treatment that consider endometriosis as an inflammatory, systemic disease that causes menstrual, cycle-dependent, chronic pain [6].

In terms of clinical management of endometriosis, surgery is still one of the first options. However, regarding the outcome from the patients’ point of view, the recurrence of the lesions and the stagnation of the symptoms are common even post-surgery. From hospitals’ perspectives, it leads to an increase in hospitalization costs [7]. Hence, long-term management options are needed that aim to alleviate pain and improve fertility. To date, the best non-invasive treatment for endometriosis, weighing the potential side effects and benefits, is hormonal management [6,8].

Hormonal therapies can be categorized as long-term or short-term treatments. Long-term options include combined hormonal contraceptives (CHCs), progestins, aromatase inhibitors, selective estrogen receptor modulators (SERMs), nonsteroidal anti-inflammatory drugs (NSAIDs), and opioids. From the above, CHCs and progestins comprise the first line of treatment due to their well-tolerable, mild side effects, safe long-term use, and low cost [9]. Short-term therapeutic options consist of gonadotropin-releasing hormone (GnRH) antagonists and GnRH analogs. So far, their menopause-like side effects—total estrogen suppression leading to severe bone loss and vasomotor dysfunctions—limit their use to a maximum of six months [6,10]. Yet, attempts have been made to extend the administration time of short-term hormonal therapies by examining the potentially increased effectivity of their combinations with CHCs and progestins [10,11].

Several comparative studies have been conducted with the aim of finding out whether combined therapies were superior to monotherapies and to identify the most effective medical treatment for endometriosis in terms of pain relief, yet clear recommendations were not made. Therefore, we aimed to compare the effectiveness of the above treatments for the different aspects of pain to identify which ones relieved it most successfully.

## 2. Methods

We reported our systematic review and network meta-analysis based on the recommendation of the PRISMA (Preferred Reporting Items for Systematic Reviews and Meta-Analyses) 2020 Statement [12]. This study followed the recommendations of the Cochrane Handbook for Systematic Reviews of Interventions, Version 6.3.8 [13]. The review protocol was registered on PROSPERO (York, UK) with registration number CRD42022374466.

### 2.1. Literature Search and Eligibility Criteria

A comprehensive search was conducted on 14 May 2023 in the following three databases: MEDLINE (via PubMed), Cochrane Library (CENTRAL), and Embase. Also, we looked for potentially unpublished trials on the clinical trials’ registry (http://clinicaltrial.gov) webpage. The main components of the search key were endometriosis, pain-relief substances, pain, and “random” for randomized control trials (RCTs). The full search key is presented in Supplementary S1. No language criteria or other filters were used.

Articles on premenopausal women who were diagnosed with endometriosis (P-Population) were included. The diagnosis of endometriosis was based on either of the following in all papers: clinical symptoms and/or imaging techniques and/or laparoscopic findings, and/or histology. The studies included used various pain medications for endometriosis (I-Intervention) compared to placebo or other drugs for endometriosis (C-Comparison). Our outcomes were the reduction in various pain aspects (overall pelvic pain, dysmenorrhea, dyspareunia) over three and six months of treatment from the initiation of treatment. (O-Outcome). Outcomes were assessed by either a visual analog scale (VAS) on a scale of 0–10 or a numerical rating scale (NRS) from 0–100. Data presented on the endometriosis-specific Biberoglu and Behrman scale were also included, with a scale of 0–3.

### 2.2. Inclusion and Exclusion Criteria

Eligible were RCTs that included premenopausal adult patients presenting with clinically suspected (symptom based and/or imaging based) and/or laparoscopically diagnosed and/or histologically confirmed endometriosis. The investigations focused on evaluating the effectiveness of various medical interventions for managing endometriosis-related pain, including but not limited to: GnRH agonists, GnRH agonists combined with add-back therapies, GnRH antagonists, GnRH antagonists combined with add-back therapies, combined hormonal contraceptives (CHCs), progestins, danazol, gestrinone, mifepristone, aromatase inhibitors, selective estrogen receptor modulators (SERMs), cyproterone, nonsteroidal anti-inflammatory drugs (NSAIDs), and opioids. Additionally, we sought studies reporting outcomes such as changes in the total score of endometriosis-associated pelvic pain, dysmenorrhea score, dyspareunia score, dyschezia score, and dysuria score.

We excluded cross-over trials, expectant management, articles with no information on the examined outcome at three or six months, single-arm studies, studies that assessed surgical interventions, or the combination of medications with surgical interventions. Studies not connected to any network were also excluded following data extraction. Detailed exclusion and inclusion criteria are presented in Supplementary Table S2.

### 2.3. Study Selection and Data Collection

EndNote X9 (Clarivate Analytics, Philadelphia, PA, USA) was utilized for the removal of duplicates, while rayyan.ai was employed for title and abstract screening, and EndNote X9 was also used for full-text selection. At each stage of selection, two independent reviewers (ÁC, DPK) screened the publications, with any disagreements resolved by a third reviewer (ÁJ).

Two authors (ÁC, DPK) independently extracted data into a predefined Excel spreadsheet (Office 365, Microsoft, Redmond, WA, USA). Data extracted from each eligible article included the following: first author, year of publication, study type, study location, number of centers involved, study design, demographic information (sample size, age), and outcome data for statistical analyses. Any discrepancies were adjudicated by a third reviewer (ÁJ). Cohen’s kappa coefficient (κ) was calculated at each step to assess interrater reliability.

### 2.4. Quality Assessment and Quality of Evidence

The quality of the articles was assessed separately by two reviewers (ÁC and ÁJ) using the risk of bias tool RoB 2 (version 2 of the Cochrane risk-of-bias tool for RCTs). Any disagreements were resolved by a third reviewer (DPK).

CINeMA (Confidence in Network Meta-Analysis) was used to evaluate the confidence in the findings of the network meta-analysis [14].

### 2.5. Data Synthesis and Analysis

Prior to network meta-analyses, network geometries of each outcome were presented by drawing a network plot to assess whether the treatments in the included studies were connected [15]. If an article examined several doses of a given drug, we selected the dose deemed most effective by the article.

All examined outcomes were continuous, therefore mean difference (MD) was calculated as the effect size measure. A common estimate for heterogeneity was assessed across the different comparisons. As anticipated, considerable between-study heterogeneity was present, a random-effects model was used to pool effect sizes. The calculation was made in a frequentist framework following the description of Harrer et al. (2021) [16]. Multi-arm study correlation was taken into consideration.

The loop-specific approach was applied to assess the presence of inconsistency. This method evaluates the consistency assumption between direct and indirect estimates for a specific comparison in each closed loop of treatments. Inconsistency was deemed acceptable if the indirect estimate—with its 95% confidence interval—for a given treatment comparison fell within the 95% confidence interval of the direct estimate of the same treatment pair.

The ranking probabilities for all treatments were also estimated to obtain some kind of treatment hierarchy for each outcome. For this, primarily p-scores were utilized. This p-score gave us the probability that among the included treatments, a given treatment ranks first. Furthermore, the surface under the cumulative ranking (SUCRA) plots were also assessed [17].

A comparison-adjusted funnel plot was generated to evaluate network-wide publication bias and the small-study effect for outcomes that included at least 10 studies within the network [18].

The results were presented on forest plots for an easier comparison of the different treatments, p-scores and SUCRA plots for the visualization of treatment ranking, netsplit plots to visualize possible inconsistencies, funnel plots for publication bias, and direct evidence plots to assess the robustness of effect size estimates within a network meta-analysis framework.

All calculations were done using the R-statistical software (version 4.2.3; R Core Team under the auspices of the R Foundation for Statistical Computing, Vienna, Austria, 2023) [19]. The following packages were used for the analyses and visualization: netmeta and BUGSnet [20,21].

## 3. Results

**Figure 1:**
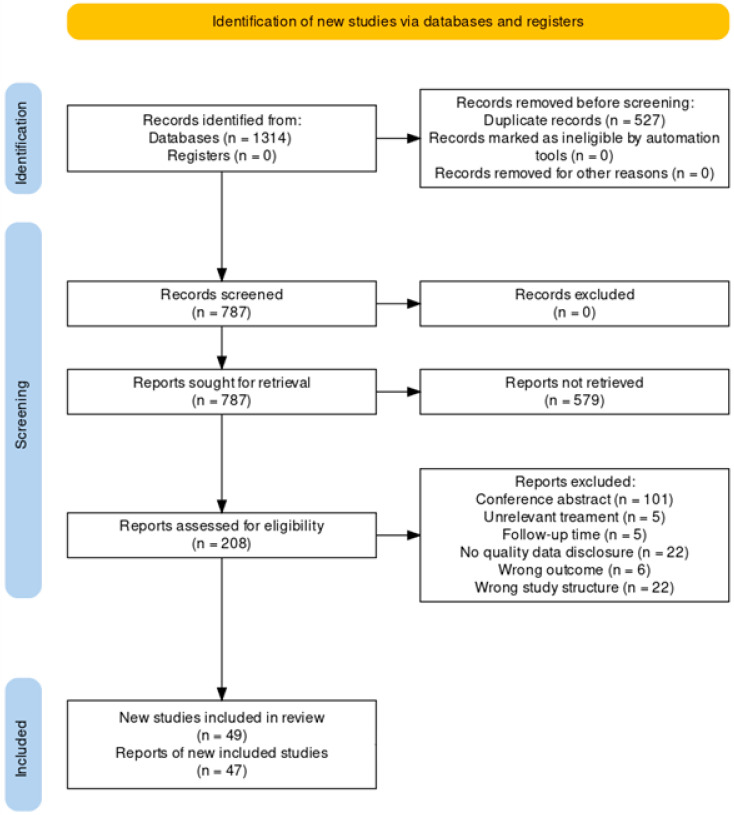
PRISMA flow diagram.

Our systematic search initially identified a total of 1314 studies. Following the removal of duplicates and the selection of relevant records, 45 studies were deemed eligible for inclusion in both qualitative and quantitative synthesis. The selection process is detailed in Figure 1.

### 3.1. Basic Characteristics

The baseline characteristics of the enrolled analyses are detailed in Supplementary Table S3. Altogether 10,529 patients were involved from 16 countries between 1987 and 2022.

### 3.2. Outcomes

Including placebo, a total of 15 treatments and treatment combinations were examined. Due to the lack of data, not all drugs could be compared with one another. The active substances and combinations of active substances were classified into 16 larger groups based on their mechanism of action, detailed in Supplementary Table S4. Our outcomes were given on a scale of 0–100 and 0–3, with higher numbers representing more intense pain. We examined three aspects of pain: overall pelvic pain, dysmenorrhea and dyspareunia. Follow-up data were available at three and six months. Integrating the above, 12 combinations were obtained for follow-up time, for pain scale, and for aspects of pain. However, dysmenorrhea on a scale of 0–3 at six months, dyspareunia on a scale of 0–3 at three and six months, and overall pelvic pain on a scale of 0–3 at six months were excluded due to an insufficient number of articles. Therefore, a total of eight networks were evaluated. Data on other aspects of pain, namely pelvic tenderness, pelvic induration, dyschezia, and dysuria, were not available for the network.

#### 3.2.1. Dysmenorrhea on a Scale of 0–100 After 3 Months

Five articles evaluated a total of six types of treatments regarding dysmenorrhea on a scale of 0–100 following three months of treatment [22,23,24,25]. The highest p-score was achieved by GnRH agonists (p-score: 0.618), deeming it to be the best option, and the lowest by placebo (p-score: 0.268). None of the drugs showed a statistically significant difference compared to placebo. Additional figures are included in the Supplementary Materials (Figures S1–S4).

#### 3.2.2. Dysmenorrhea on a Scale of 0–3 After 3 Months

Three articles evaluated a total of four types of treatments regarding dysmenorrhea on a scale of 0–3 following three months of treatment [10,26,27]. The highest p-score was achieved by GnRH agonists (p-score: 0.828) and the lowest by placebo (p-score: 0.145). None of the drugs showed a statistically significant difference compared to placebo. Additional figures are included in the Supplementary Materials (Figures S5–S8).

#### 3.2.3. Dysmenorrhea on a Scale of 0–100 After 6 Months

**Figure 2:**
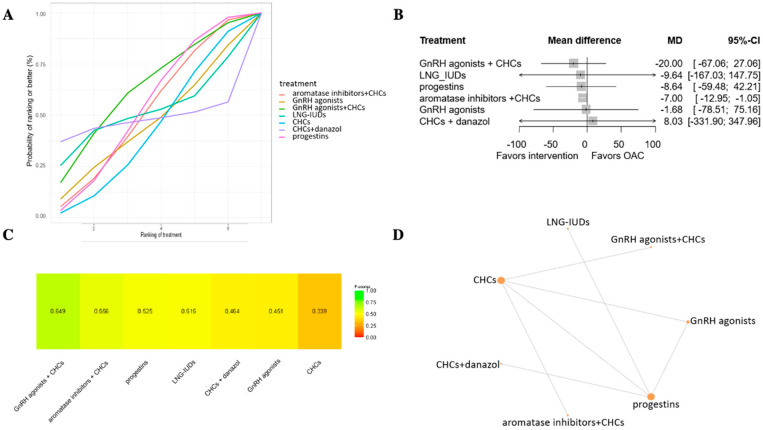
Treatment options with the possibility of being ranked from best to worst efficacy in dysmenorrhea on a scale of 0–100 after 6 months. (**A**) Probability chart showing likelihood in percentage of treatments being ranked from best to worst based on the SUCRA values. (**B**) Forest plot showing calculated mean difference (MD) with its 95% confidence interval. (**C**) The analysis shows the probability of all interventions to match the top rank with a numerical representation of the SUCRA. The closer to 1 the SUCRA value is, the higher the likelihood that a therapy is in the top rank; the closer to 0 it is, the more likely that a therapy is in the bottom rank. (**D**) Network plot of randomized controlled trials comparing different treatment options. CHC = combined hormonal pill with estrogen and progesterone.

Ten articles evaluated a total of seven types of treatments regarding dysmenorrhea on a scale of 0–100 following six months of treatment [11,23,28,29,30,31,32,33,34,35]. The highest p-score was achieved by GnRH agonists combined with CHCs (p-score: 0.649) and the lowest by CHCs (p-score: 0.339) (Figure 2). For dysmenorrhoea, there were no articles available that examined placebo; therefore, CHCs were chosen as references. Neither drug showed a statistically significant difference compared to CHCs.

#### 3.2.4. Dyspareunia on a Scale of 0–100 After 3 Months

Seven articles evaluated a total of seven types of treatments regarding dyspareunia on a scale of 0–100 following three months of treatment [22,23,24,32,33,36,37]. The highest p-score was achieved by CHCs (p-score: 0.805), and the lowest by placebo (p-score: 0.381). None of the drugs showed a statistically significant difference compared to placebo. Additional figures are included in the Supplementary Materials (Figures S9–S12).

#### 3.2.5. Dyspareunia on a Scale of 0–100 After 6 Months

Eleven articles evaluated a total of eight types of treatments regarding dyspareunia on a scale of 0–100 following six months of treatment [11,23,29,31,32,33,34,35,38,39]. The highest p-score was achieved by CHCs combined with aromatase inhibitors (p-score: 0.677) and the lowest by SERMs (p-score: 0.315). None of the drugs showed a statistically significant difference compared to placebo. Additional figures are included in the Supplementary Materials (Figures S13–S16).

#### 3.2.6. Overall Pelvic Pain on a Scale of 0–100 After 3 Months

A total of 15 articles evaluated 7 types of treatments regarding overall pelvic pain on a scale of 0–100 following three months of treatment [22,27,36,40,41,42,43,44,45,46,47,48,49,50,51]. Compared to placebo, only GnRH agonists and antagonists showed a statistically significant difference; however, CHCs (p-score: 0.751) received the highest p-score and placebo (p-score: 0.179) the lowest. Additional figures are included in the Supplementary Materials (Figures S17–S20).

#### 3.2.7. Overall Pelvic Pain on Scale of 0–3 After 3 Months

A total of three articles evaluated four types of treatments regarding overall pelvic pain on a scale of 0–3 following three months of treatment [10,52,53]. Compared to placebo, progestins (p-score: 0.901) showed a statistically significant difference; they also achieved the highest p-score, and GnRH antagonists (p-score: 0.257) got the lowest. Additional figures are included in the Supplementary Materials (Figures S21–S24).

#### 3.2.8. Overall Pelvic Pain on Scale of 0–100 After 6 Months

**Figure 3:**
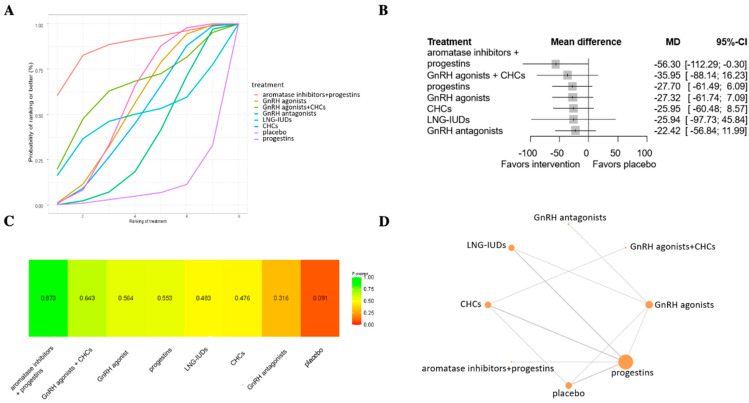
Treatment options with the possibility of being ranked from best to worst efficacy in overall pelvic pain on a scale of 0–100 after 6 months. (**A**) Probability chart showing likelihood in percentage of treatments being ranked from best to worst based on the SUCRA values. (**B**) Forest plot showing calculated mean difference (MD) with its 95% confidence interval. (**C**) The analysis shows the probability of all interventions to match the top rank with a numerical representation of the SUCRA. The closer to 1 the SUCRA value is, the higher the likelihood that a therapy is in the top rank; the closer to 0 it is, the more likely that a therapy is in the bottom rank. (**D**) Network plot of randomized controlled trials comparing different treatment options. CHC = combined hormonal pill with estrogen and progesterone.

Twenty-one articles evaluated a total of eight types of treatments regarding overall pelvic pain on a scale of 0–100 following six months of treatment [29,30,31,38,39,43,44,45,48,50,51,54,55,56,57,58,59,60,61,62,63]. The highest p-score was given to progestins combined with aromatase inhibitors (p-score: 0.873), and the lowest to placebo (p-score: 0.091) (Figure 3). None of the drugs showed a statistically significant difference compared to placebo.

### 3.3. Quality and Risk of Bias Assessment

The risk of bias was assessed using the RoB 2 tool. Most articles were deemed to be of low risk. The ones receiving a high overall risk lacked information on blinding (Supplementary Table S5).

After completing the quality control process, the findings revealed that in the majority of cases, a very low rating was observed, and in some cases, a low rating was noted (Supplementary Table S6).

## 4. Discussion

This systematic review and meta-analysis identified 45 studies that examined the drugs involved in pain relief associated with endometriosis. The quantitative synthesis of our findings proved GnRH antagonists to be the most effective for dysmenorrhea, CHCs in the treatment of dyspareunia, and for overall pelvic pain, CHCs or progestins combined with aromatase inhibitors.

The first network meta-analysis examining treatment options for patients with endometriosis was published in 2019, and it showed that expectant management, progestins, and GnRH agonists were effective in the reduction of pain when compared with placebo. Yet, despite thorough analysis, no clear conclusion was reached that would have deemed either pharmaceutical or surgical intervention to be more effective over another [64]. In 2020, Samy et al. published another meta-analysis ranking dienogest, combined hormonal contraceptives (CHCs), and elagolix as drugs of highest efficacy in the reduction of pelvic pain following three months. Following six months, the highest rankings were given to GnRH agonists, LNG-IUDs, and dienogest. Furthermore, based on the overall ranking, the most effective medical treatments were GnRH agonists and CHCs in the reduction in dysmenorrhoea-associated pain [65]. The most recent guidelines from the French National Authority for Health (HAS) and the French College of Gynecologists and Obstetricians (CNGOF) recommend CHCs and LNG-IUDs as the primary options for managing pain associated with endometriosis [66].

Regarding dysmenorrhea, it can be declared that, during a 3-month follow-up period, GnRH agonists emerged as the most effective treatment. Since GnRH agonists induce secondary amenorrhea, in the absence of menstrual bleeding there is a reduction in discomfort. Moreover, for the 6-month follow-up, our findings suggest that GnRH agonists paired with combined hormonal contraceptives (CHCs) should be prioritized as the primary therapy. Consequently, based on these results, it is advisable in routine clinical practice to complement initiated GnRH agonist treatment with a CHC after the initial 3 months.

In the context of dyspareunia, it is evident that combined hormonal contraceptives (CHCs) were consistently the preferred therapeutic option throughout all 3-month follow-up periods. Concerning the 6-month follow-up period, the combination of CHCs with aromatase inhibitors demonstrated the highest effectiveness, albeit without a significant difference compared to the most effective 3-month therapy, which constitutes CHCs. Consequently, in practical terms, CHCs remain the primary choice for the investigated 6-month observation period.

Concerning overall pelvic pain, at the 3-month follow-up period, combined hormonal contraceptives (CHCs) received the highest p-score on a scale of 0–100. It is important to note, however, that only GnRH agonists and antagonists demonstrated a significant difference compared to the placebo. Nevertheless, when interpreting the collective results, CHCs should be prioritized as the primary choice of therapy. Conversely, on a scale of 0–3, progestins proved to be the most effective, displaying the sole significant difference. Notably, only three studies assessed this outcome on the 0–3 scale, while 16 studies implemented the 0–100 scale. Therefore, based on the findings from studies with the 0–100 scale, CHCs are recommended in clinical practice as the preferred therapeutic option. For the 6-month follow-up period, progestins combined with aromatase inhibitors emerged as the most effective treatment. As there is no available 3-month follow-up data for progestins combined with aromatase inhibitors, direct comparison with CHCs is challenging. Consequently, both CHCs and progestins combined with aromatase inhibitors can be suggested as therapies to alleviate overall pelvic pain.

Certain medications commonly used in clinical practice, such as nonsteroidal anti-inflammatory drugs (NSAIDs), were not investigated in the eligible articles. NSAIDs have been used for decades in pain management, particularly for dysmenorrhea. However, the assessment of available literature suggests that their use is more influenced by clinical practice than evidence-based decision-making [67]. A cyclooxygenase-2 inhibitor, rofecoxib, was also being tested in 2004 for the reduction of endometriosis-associated pain, but since then it has been withdrawn from the market [68]. A straightforward option for pain management would be the utilization of opioids. However, no clinical trials have explored their effectiveness in reducing endometriosis-associated pain, and they were not mentioned as a potential option in the 2022 European Society of Human Reproduction and Embryology (ESHRE) Guideline [69].

### 4.1. Strengths and Limitation

Regarding the strengths of our analysis, the selected articles enrolled a total of approximately 10,000 patients. We would also like to emphasize the good study design and the high quality of the articles included. Our network meta-analysis was performed and documented in accordance with the established guidelines outlined in the Cochrane Handbook and the PRISMA-NMA statements. Some limitations of our study should be mentioned. There was variability in the diagnosis of endometriosis across the included studies, contributing to increased heterogeneity. Significant improvements in the accuracy of estimates could be achieved if the diagnosis of endometriosis were standardized. In certain treatment arms, no robust conclusions could be drawn due to the limited number of available studies. The grouping of individual drugs or drug combinations based on broad drug categories may obscure distinctions between specific types of medications. Additionally, there was a notable risk of bias in specific domains of the randomized controlled trials (RCTs) included.

### 4.2. Implication for Research and Practice

First and foremost, dyschezia and dysuria need to be further investigated, as they represent two of the most prominent symptoms of deeply infiltrating endometriosis for which there is currently insufficient data available. Additional RCTs would be needed for those drug types where fewer studies have been conducted. Overall, it can be said that a study with longer observation periods would be necessary, as it would be useful to see the effect of these drugs over a period of years. Future research could yield more precise results by consistently defining endometriosis diagnosis, with the current gold standard being histopathological confirmation obtained during surgery. Furthermore, in practice, the attending physician must, together with the patient, choose the treatment that best suits the patient, and the ranking we have determined helps in this complicated matter.

## 5. Conclusions

Translating scientific knowledge for patient benefit has crucial importance [70,71]. Our network meta-analysis showed that in cases of dysmenorrhea, GnRH agonists supplemented with CHCs after 3 months, in cases of dyspareunia, CHCs alone, and in cases of overall pelvic pain, CHCs or progestins combined with aromatase inhibitors should be the first choice for the management of endometriosis-related pain.

## Data Availability

The datasets used in this study can be found in the full-text articles included in the systematic review and meta-analysis.

## Supplementary Materials

The following supporting information can be downloaded at: https://www.mdpi.com/article/10.3390/jcm13226932/s1.
