# Supplementary material for: Comparative Analysis of Medical Interventions to Alleviate Endometriosis-Related Pain: A Systematic Review and Network Meta-Analysis
Source: J Clin Med. 2024 Nov 18;13(22):6932. doi: 10.3390/jcm13226932 (PMC11595017; doi:10.3390/jcm13226932)
Supplement: Supplementary file 1 [file jcm-13-06932-s001.zip › jcm-3325212-supplementary.pdf]

Supplementary Material

Comparative Analysis of Medical Interventions to Alleviate Endometriosis-Related Pain: A Systematic Review and Network Meta-Analysis

Ádám Csirzó [1,2], Dénes Péter Kovács [1,2], Anett Szabó [1,3], Bence Szabó [1], Árpád Jankó [1], Péter Hegyi [1,4,5], Péter Nyirády [1,3], Nándor Ács [1,2] and Sándor Valent [1,2,*]

[1] Centre for Translational Medicine, Semmelweis University, 1085 Budapest, Hungary; adamcsirzo@gmail.com (Á.C.); kovacsdenespeter@gmail.com (D.P.K.); a.szabo1995@gmail.com (A.S.); bencetra@gmail.com (B.S.); jankoarpi@gmail.com (Á.J.); hegyi2009@gmail.com (P.H.); nyiradyp@gmail.com (P.N.); acsnandor@gmail.com (N.Á.)
[2] Department of Obstetrics and Gynecology, Semmelweis University, 1082 Budapest, Hungary
[3] Department of Urology, Semmelweis University, 1082 Budapest, Hungary
[4] Institute of Pancreatic Diseases, Semmelweis University, 1083 Budapest, Hungary
[5] Institute for Translational Medicine, Medical School, University of Pécs, 7624 Pécs, Hungary
* Correspondence: valent.sandor@gmail.com; Tel.: +3630-3147757

TABLE OF CONTENT

Table S1. Detailed exclusion and inclusion criteria of the included articles
Table S2. Baseline characteristics table
Table S3. Final active ingredient groups following merging of substances
Table S4. Risk of bias assessment
Table S5. CINeMA grading assessment

FIGURE OF CONTENT

Figure S1. Network figure of dysmenorrhea on a scale of 0–100 after 3 months
Figure S2. Ranking probability of dysmenorrhea on a scale of 0–100 after 3 months
Figure S3. Forest plot of dysmenorrhea on a scale of 0–100 after 3 months
Figure S4. Sucra plot of dysmenorrhea on a scale of 0–100 after 3 months
Figure S5. Network figure of dysmenorrhea on a scale of 0–3 after 3 months
Figure S6. Ranking probability of dysmenorrhea on a scale of 0–3 after 3 months
Figure S7. Forest plot of dysmenorrhea on a scale of 0–3 after 3 months
Figure S8. Sucra plot of dysmenorrhea on a scale of 0–3 after 3 months
Figure S9. Network figure of dyspareunia on a scale of 0–100 after 3 months
Figure S10. Ranking probability of dyspareunia on a scale of 0–100 after 3 months
Figure S11. Forest plot of dyspareunia on a scale of 0–100 after 3 months
Figure S12. Sucra plot of dyspareunia on a scale of 0–100 after 3 months

Figure S13. Network figure of dyspareunia on a scale of 0–100 after 6 months
Figure S14. Ranking probability of dyspareunia on a scale of 0–100 after 6 months
Figure S15. Forest plot of dyspareunia on a scale of 0–100 after 6 months
Figure S16. Sucra plot of dyspareunia on a scale of 0–100 after 6 months
Figure S17. Network figure of overall pelvic pain on a scale of 0–100 after 3 months
Figure S18. Ranking probability of overall pelvic pain on a scale of 0–100 after 3 months
Figure S19. Forest plot of overall pelvic pain on a scale of 0–100 after 3 months
Figure S20. Sucra plot of overall pelvic pain on a scale of 0–100 after 3 months
Figure S21. Network figure of overall pelvic pain on a scale of 0–3 after 3 months
Figure S22. Ranking probability of overall pelvic pain on a scale of 0–3 after 3 months
Figure S23. Forest plot of overall pelvic pain on a scale of 0–3 after 3 months
Figure S24. Sucra plot of overall pelvic pain on a scale of 0–3 after 3 months

APPENDIX OF CONTENT

Supplementary S1. Search key

Table S1. Detailed exclusion and inclusion criteria of the included articles.

Article	Inclusion criteria	Exclusion criteria
Vercellini – 1994	• women aged 18–45 years, who have been diagnosed with endometriosis by laparoscopy	• treatment for endometriosis (other than NSAIDs) in 3 mts before study entry<br>• any contraindications for danazol<br>• unwillingness to use barrier method as contraception
Vercellini – 2005	• women aged 18–35 years with normal menstrual cycles and with stage I–IV endometriosis diagnosed by laparoscopy, without the presence of rectovaginal lesions	• obstructive uropathy<br>• bowel stenosis<br>• complex adnexal cysts<br>• ovarian endometrioma ≥3 cm<br>• treatment for endometriosis (other than NSAIDs) in 3 mts before study entry<br>• CI for E2 and P<br>• known allergic reactions to constituents of galenical formulations or to nonsteroidal anti-inflammatory drugs<br>• unwillingness to tolerate menstrual changes<br>• a diagnosis of concomitant pelvic inflammatory disease, pelvic varices, or genital malformations at previous surgery<br>• known gastrointestinal, urologic, and orthopedic diseases<br>• psychiatric disturbances<br>• history of drug or alcohol abuse.
Ács – 2015	• women aged 18–45 years with the diagnosis of endometriosis by laparoscopy within 60 weeks of enrolment, who had total Composite Pelvic Signs and Symptoms Score (CPSSS) ≥6, with a score of ≥2 for dysmenorrhea and a score of ≥1 for non-menstrual pelvic pain	• Patients were excluded if they were administered a GnRH agonist or antagonist, or danazol within 6 months of screening,<br>• depot medroxyprogesterone acetate within 3 months of screening<br>• used hormonal contraception or other hormonal therapy within 1 month of screening<br>• if they had a history of unresponsiveness to GnRH agonist or antagonist treatment
Taylor – 2017	• women aged 18–49 years, who had received their surgical diagnosis of endometriosis within 10 years of	• having a Z score of less than -1.5 for bone mineral density at the lumbar spine, femoral neck or total hip at screening

	study enrolment and had severe to moderate endometriosis associated pain	• clinically significant other gynecologic condition or chronic pain unrelated to endometriosis
Donnez - 2020	• women aged 18–45 years with surgical diagnosis of endometriosis within 10 years of enrolment, who had been experiencing moderate or severe endometriosis-associated pain as symptoms at study baseline	• chronic pelvic pain was not caused by endometriosis<br>• they had liver enzyme anomalies<br>• osteoporosis<br>• other metabolic bone disease<br>• oral contraceptives<br>• GnRH analogs<br>• systemic glucocorticoids, with a specified wash-out period<br>• not accepting the use of non-hormonal barrier contraception
Xue – 2016	• women who had been diagnosed endometriosis by laparoscopy or 3D ultrasound	• pregnancy<br>• lactation<br>• drug allergy<br>• CI for mifepristone or gestrinone<br>• cardiorespiratory disfunction<br>• hepatic dysfunction<br>• renal disfunction<br>• endocrine diseases
Soysal – 2004	• women with severe endometriosis based on the revised American Reproductive Medicine classification (rASRM<40)	• further desire for childbearing<br>• any treatment for endometriosis within the previous 3 months<br>• any concomitant disease that can be an established cause of chronic pelvic pain<br>• osteopenia or osteoporosis<br>• CI for gosereline or anastarazol
Stratton – 2008	• women aged 18–45 years presenting with at least 3 months of pelvic pain caused by biopsy-proven endometriosis with significant reduction of post-operative pelvic pain	• BMI>40<br>• use of antidepressant, medication for migraines, headaches, allergy medication<br>• refusal of using non-hormonal contraception
Osuga – 2021	• premenopausal Japanese females who had completed treatment with relugolix in phase 2 study	• who had treatment-emergent adverse events for relugolix<br>• inability to comply with study protocol<br>• who were unresponsive to treatment in phase 2<br>• who showed symptoms of occurrence of hypoestrogenism

Guzick – 2011	• women aged 18–menopause with symptoms of moderate to severe pelvic pain for at least a period of 18 months before enrolment caused by endometriosis that had been surgically and histologically proven by within 3 years of study enrolment with the willingness to comply with study protocol.	• use of oral contraceptives within 1 month of enrollment<br>• dose of leuprolide within 3 months if given monthly or within 5 months if given 3-month injection<br>• any CI for OC use (including preexisting conditions and lifestyle attributes)<br>• history of hysterectomy and bilateral salpingo-ophorectomy<br>• pregnancy or breastfeeding.<br>• significant mental or chronic disease that might influence the ability of pain assessment or completing the study
Caruso – 2022	• women aged 18–39 years with chronic pelvic pain, dysmenorrhea and dyspareunia, whose CPP symptoms had lasted for a period of 2-10 years and who had been using NSAIDs for a period of 15 months–8 years	• on GnRH or other hormonal treatments within the past 3-6 months<br>• affected by infertility<br>• women in asexual relationships or with sexual disfunction
Tekin – 2011	• women aged 18–45 years with a histologically and surgically proven endometriosis and chronic pelvic pain	• infertility<br>• current breastfeeding<br>• desire for pregnancy<br>• any hormonal therapy within 3 months of trial<br>• history of pelvic inflammatory disease<br>• having uterine and adnexal pathologies other than endometriosis<br>• CI for the use of IUS
Tanmahasamut – 2017	• women with moderate to severe dysmenorrhea and/or pelvic pain that had lasted for a period of more than 6 months and who had been already scheduled for laparoscopy	• pelvic pain caused by other than endometriosis<br>• had uterine or adnexal anomalies other than endometriosis<br>• had current treatments for endometriosis other than analgesic medications<br>• were unable to undergo conservative surgery<br>• had CI for desogestrel<br>• unable to tolerate menstrual changes<br>• planned pregnancy in 6 months<br>• refusal to participation
Cheewadhanaraks – 2012	• women aged 18–40 years with endometriosis-associated pain for a period of at least 6 months, who	• endometriosis treatment other than NSAIDs in the past 6 months

	did not plan pregnancy in the following 18 months at study baseline	• other pelvic pathologies other than endometriosis<br>• known gastrointestinal, urologic or orthopedic diseases or CI for DMPA or OC
Telimaa – 1987	• women with laparoscopic diagnosis of endometriosis	• N/A
Giudice – 2022	• women aged 18–50 years with laparoscopically visualized endometriosis with or without histological diagnosis within the past 10 years of enrolment	• bone mineral density by dual energy X-ray absorptiometry Z score of less than -2.0 at the lumbar spine, total hip or femoral neck<br>• history of chronic pain not caused by endometriosis<br>• having any contraindication to hormonal therapy
Harada – 2008	• women aged 18 years and older with regular menstrual cycles, with a laparoscopically diagnosed endometriosis or endometrioma, who had normal cervical and endometrial smear cytology with moderate or severe dysmenorrhea and who had used no hormonal or surgical treatment for endometriosis within 8 weeks of study enrolment	• contraindication for OC use<br>• any issues that do not meet the inclusion criteria
Zhao – 2020	• reproductive aged women with cyclic, predictable menses with at least one kind of endometriosis pain present and who were diagnosed by laparoscopy and whose pain was refractory after previous treatment	• undiagnosed vaginal bleeding<br>• endometriomas >2 cm<br>• sensitivity to letrozole<br>• seizure disorders<br>• pregnancy<br>• pulmonary, cardiac, renal, or hepatic diseases
Vercellini - 1996	• women between the ages of 18 and 40 with chronic pelvic pain who did not want pregnancies in the near future of the study baseline and who had been diagnosed with endometriosis by laparoscopy and had not had any surgical or medical treatments for endometriosis	• treatment for endometriosis other than nonsteroid anti-inflammatory drugs in the past 6 months prior to the study<br>• concomitant disorder other than endometriosis that can cause gynecologic pain<br>• contraindication for use of GnRH agonists<br>• abnormal baseline mineral bone density values<br>• unwillingness to use barrier contraception
Harada – 2017	• women aged 20 years or older with clinical diagnosis of endometriosis who had pelvic tenderness, induration in the cul de sac, or uterine immobility or patients with laparoscopic diagnosis of endometriosis or endometriomas	• patients who undergone surgical treatment for endometriosis within 2 months prior to the study

Margatho – 2020	• women aged between 18 and 45 who did not wish to conceive within the following 6 months and who had been diagnosed surgically or histologically with endometriosis and who had a history of dysmenorrhea or chronic pelvic pain lasting >6 months and had a VAS pain score ≥4	• unwillingness to use neither the implant nor the intrauterine system<br>• contraindications for either the ENG implant or LNG-IUS<br>• who had undergone surgical or hormonal treatment for endometriosis in 2 months preceding enrolment
Diamond – 2014	• women aged between 18 and 49 diagnosed with endometriosis by laparoscopy within 8 years of screening and had a total composite pelvic signs and symptoms score (CPSSS) ≥ 6 and scored at least moderate (≥2) for dysmenorrhea and at least mild (≥1) for non-menstrual pelvic pain at baseline and had to agree to use non-hormonal contraception during the study	• if patients had been using GnRH agonist, GnRH antagonist or danazol within 6 months of screening<br>• had used hormonal contraception or other hormonal therapy within 1 month of screening<br>• had history of unresponsiveness to GnRH agonist or antagonist therapy<br>• had surgical treatment for endometriosis within 1 month of screening
Harrison – 2000	• women aged between 20 and 39 with history of infertility (at least 2 years) and who were diagnosed with endometriosis by laparoscopy, and where no other potential cause of infertility was found (e.g. semen analyses) and were willing to participate in the study	• uterine, ovarian or tubule defect other than endometriosis<br>• history of cancer<br>• renal or hepatic impairment<br>• diabetes mellitus<br>• history of treatment that might influence endometriosis within 4 weeks of baseline laparoscopy
El Taha – 2021	• women aged between 20 and 45 with histologically confirmed endometriosis or diagnosis of deep endometriosis and/or ovarian endometrioma via imaging studies with complaints of dysmenorrhea, non-cyclic chronic pelvic pain or both for a period of at least 6 months, with regular menstrual cycles, with endometriosis-associated pelvic pain scoring ≥4 on VAS at baseline, with the presence of one or more subjective symptoms for endometriosis during menstruation (e.g., lower abdominal pain, lumbago,	• undiagnosed genital bleeding and/or other abnormal findings on gynecological examination other than endometriosis<br>• use of hormonal therapy for endometriosis 16 months before enrollment<br>• pregnancy, lactation, or desire for pregnancy during treatment period<br>• previously failed treatment for endometriosis using medications including in the study<br>• history of severe drug reaction or hypersensitivity to steroid hormones

	etc.) and with the presence of one or more subjective symptoms of endometriosis outside menstruation	• contraindications to COC or dienogest<br>• prior surgical treatment or examination for endometriosis within a menstrual cycle before the start of the medication
D'Hooghe – 2019	• women aged 18 to 45 years with moderate to severe endometriosis causing dysmenorrhea and non-menstrual pelvic pain with regular menstrual cycle of 24–35 days who had been diagnosed laparoscopically.	• treatments that alter gynecological endocrinology or surgery for endometriosis within 4 weeks of study initiation<br>• presence of pelvic or gynecological abnormalities
Bergqvist – 1998	• women aged 19–44 years who had been menstruating regularly (25–35 days interval) for at least 3 months before study, had clinical symptoms of endometriosis	• oral contraceptives use or any oral steroid therapy 3 months prior to the study<br>• use of long-lasting depot gestagens or GnRH agonists within the preceding 6 months<br>• breastfeeding or pregnancy during and within the preceding 3 months of study<br>• history of osteoporosis and coagulation disorders
Ferreira – 2009	• women aged 18–40 years with laparoscopic or histologic diagnosis of endometriosis and presented with chronic pelvic pain who had not been treated either with oral contraceptives for at least 3 months or with depot progestogens or GnRH agonists for at least 6 months at the time of randomization	• obese patients with a BMI $\geq 30 kg/m^2$<br>• smokers<br>• diabetics<br>• alcohol or drug users<br>• patients who wish to conceive<br>• patients with chronic disease or with acute or chronic inflammatory and/or infectious processes<br>• patients with a personal and/or family history of thromboembolic events

		• taking any medication that is known to interfere with inflammation markers for a period of less than 15 days before the study
Ashraf – 2022	• women aged 18-60 years with endometriosis baseline pain >5 at VAS scale	• endometrioma >3 cm<br>• uropathy<br>• endometriotic nodules infiltrating muscular layer of the bowel wall<br>• receiving treatment for endometriosis other than NSAIDs in the last 6 months<br>• seizure disorder<br>• osteopenia<br>• pregnancy
Abdou – 2018	• women aged 20–45 years with recurrent pelvic pain who had been diagnosed with endometriosis by diagnostic laparoscopy within 3 months of study or by therapeutic laparoscopy within 12 months of enrollment	• pregnancy<br>• breastfeeding<br>• amenorrhea within 3 months of study<br>• previous use of hormonal agents following laparoscopy<br>• undiagnosed genital bleeding<br>• history of severe adverse drug reaction or hypersensitivity to steroid hormones or GnRH agonists<br>• history of embolism/thrombosis<br>• depression<br>• patients at risk of decreased bone mineral density

Strowitzki – 2010	• women aged 18–45 years with pain associated with histologically proven endometriosis that was confirmed by diagnostic laparoscopy within 3 months or therapeutic laparoscopy within 12 months of study	• pregnancy<br>• breastfeeding<br>• amenorrhea within 3 months of study<br>• primary need for surgical treatment for endometriosis<br>• previous use of hormonal agents<br>• abnormal findings at gynecological examination<br>• abnormal cervical cytological smear in the last 3 months<br>• risk factors for decreased bone mineral density
Harada – 2009	• women aged 20 or older with regular menstrual cycles who have endometriosis diagnosed by laparoscopy or imaging analyses of endometriotic ovarian chocolate cysts with the presence of subjective symptoms during menstruation and non-menstruation and with objective findings (e.g., limited uterine mobility)	• undiagnosed genital bleeding<br>• class 3 or higher Pap smear in the last 3 months<br>• use of GnRH agonists, testosterone derivatives, hormonal therapy with progesterone and/or estrogen, estrogen antagonists or aromatase inhibitors within 16 weeks of enrollment<br>• pregnancy or nursing<br>• history of severe adverse drug reaction or hypersensitivity to steroid hormone or GnRH agonists<br>• past use of GnRH agonists with low bone mineral density<br>• having undergone surgery therapy or surgical examination for endometriosis within a menstrual cycle before the start of medication<br>• use of drugs that could be expected to affect the release of sex hormones (e.g., sulpiride, cimetidine)<br>• a history or complication of thrombosis/embolism or depression<br>• malignant tumor complication or findings suggestive of a malignant tumor<br>• complication of serious heart, liver, kidney, blood, or endocrine disease<br>• participation in another clinical trial within the 4 months before enrollment<br>• patients deemed unsuitable for study entry by the investigator

Strowitzi – 2010	• women aged between 18 and 45 years between menarche and menopause who are in good general health, with or without infertility who received a histologically proven endometriosis diagnosis by laparoscopy within 12 months of study baseline and who had an EEAP score $\geq$30mm on a visual analogue scale	• amenorrhea for 3 or more months<br>• primary need for surgical treatment for endometriosis<br>• previous use of hormonal agents within 1–6 months of screening<br>• abnormal findings on gynecological examination other than endometriosis
Lang – 2017	• women aged 18–45 years with diagnosis of endometriosis by laparoscopy within 10 years of study entry who had a VAS score >30 mm for EAPP over the past 4 weeks at the screening visit	• pregnancy or lactation<br>• planning of pregnancy<br>• amenorrhea for 3 or more months in the last 6 months<br>• undiagnosed genital bleeding<br>• evidence for therapy resistant endometriosis<br>• recent use of hormonal agents<br>• required surgical treatment for endometriosis
Takaesu – 2016	• women aged 18–pre-menopause with endometriosis diagnosis within 3–7 years of enrollment	• hormonal therapies (GnRH agonists, danazol, LEP, dienogest, and progestin) within 6 months before the surgery
Strovitzki – 2012	• women aged 18–45 years with de novo or recurrent pain associated with a confirmed diagnosis of endometriosis	• amenorrhea >3 months<br>• need for surgical treatment for endometriosis<br>• previous use of hormonal treatments within specified times<br>• abnormal findings on gynecological examination<br>• pregnancy or breastfeeding<br>• risk factors for decreased bone mineral density

Vercellini – 1996	• women aged 18–40 years with a diagnosis of endometriosis by laparoscopy with no attempts at implant reduction within 3 months before enrollment and who had pelvic pain of >6 months' duration	• treatment for endometriosis other than NSAIDs within 3 months of study entry<br>• contraindications for estrogen, progestins and danazol<br>• unwillingness to tolerate menstrual changes<br>• wish to conceive in the following 2 years
Vercellini – 2002	• women aged 18–40 years who did not wish to conceive and had first-line conservative surgery at laparoscopy in the previous 12 months	• therapies for endometriosis other than NSAIDs within 6 months of enrollment<br>• contraindications for estrogen and progestins<br>• unwillingness to tolerate menstrual changes<br>• diagnosis of concomitant pelvic inflammatory disease<br>• varices or genital malformations at previous surgery<br>• known gastrointestinal, urologic or orthopedic diseases<br>• psychiatric disturbances<br>• evidence of complex adnexal cysts or of an ovarian endometrioma $\geq$3 cm in diameter at vaginal ultrasonography
Carvalho – 2018	• women aged 18–45 years with a diagnosis of endometriosis by laparoscopy or transvaginal ultrasonography and MRI who had complaints of non-cyclic chronic pelvic pain and dysmenorrhea or both for more than 6 months and who were clinically healthy and were able to keep a menstrual diary and were willing to return to follow-up visits and agreed to participate in the study	• pregnancy<br>• contraindications of ENG implant or LNG-IUS defined by the World Health Organization<br>• women who had undergone surgical treatment for endometriosis within 2 months of enrollment

Niakan – 2021	• women aged 18–45 years who had endometriosis diagnosed by laparoscopy and had a BMI 18,5-24,9 kg/m2, presented with subjective symptoms during menstruation, had no pelvic pain originating from other organs including gastrointestinal, genitourinary systems, had no use of gonadotropin analog treatment or other hormonal drugs in the last 3 months, had no gynecological malignancy, had no other gynecological disease along with endometriosis (such as non-endometrioid ovarian cyst), had no underlying diseases such as cardiovascular, respiratory, renal, hematologic, hepatic, neurologic, or psychological disorders, had no contraindications to OCPs or dienogest use, had no plan for pregnancy in near future	• high risk of thrombosis<br>• any gynecological disease other than endometriosis<br>• pregnancy<br>• unwillingness to cooperate<br>• history of adverse drug reaction or hypersensitivity to steroid hormones<br>• participation in research with interventions in a common clinical procedure
Margatho – 2018	• women aged 18–45 years who had a diagnosis of endometriosis by laparoscopy or histologically with endometriosis-associated pain with a VAS score > 4	• pregnancy<br>• desire to conceive within 12 months<br>• contraindication for the use of ENG implants or LNG-IUS determined by WHO<br>• surgery for endometriosis within 2 months of study<br>• use of hormonal treatment during the study
Zupi – 2004	• women aged 20–43 years, with regular menstrual cycles and a history of symptomatic severe endometriosis diagnosed surgically and presented with recurrent pelvic pain, dysmenorrhea and dyspareunia	N/A

Kashi – 2011	• women aged 18–45 years with a body mass index 18,5–29,9 kg/m2, presented with stage IV endometriosis based on the rASRM classification, with subjective symptoms during menstruation without any originating source of pelvic pain, who had not used GnRH analog or any other hormonal treatment in the last 3 months before the study, who had no gynecological malignancy or no other gynecological disease other than endometriosis that was confirmed by ultrasound and MRI, who had no underlying diseases such as cardiovascular, respiratory, hematologic, hepatic, neurologic or psychological condition and who had no contraindications to COCP or dienogest	• desire for pregnancy in the near future or pregnancy<br>• any other gynecological diseases confirmed by surgery or pathological reports<br>• required bladder or rectum resection<br>• unwillingness to participate<br>• severe adverse drug reaction or hypersensitivity to a steroid hormone<br>• participation in in an investigational program with interventions outside of routine clinical practice
Vercellini – 1993	• women aged 18–35 years with endometriosis diagnosed by laparoscopy while there were no attempts at endometriosis reduction within 3 months of study entry	• receiving any treatments for endometriosis other than NSAIDs in the preceding 3 months before trial<br>• any contraindications to oral contraceptives

Table S2. Baseline characteristics table. OP: surgery-based diagnosis, SY: symptoms-based diagnosis, IM: imaging-based diagnosis, na: no data.

First Author	Year of publication	Study period	Population	Diagnosis	Types of drugs compared	Total no. of patient	Other
Abdou, A.M.	2018	May 2014 to December 2016	aged 20 to 45 years	OP,SY	progestins / GnRH agonists	284	
Ács, N.	2015	November 2008 to February 2011	aged 18 to 45 years	OP	GnRH antagonists / GnRH agonists / placebo	174	
Ashraf, S.	2022	February 2020 to January 2021	aged 18 to 60 years	SY	progestins / aromatase inhibitors + progestins	72	
Bayoglu Tekin, Y.	2011	2005–2007	aged 18 to 45 years	OP,SY	LNG-IUDs / GnRH agonists	40	
Bergqvist, A.	1998	na	aged 19 to 44 years	OP	GnRH agonists / placebo	49	
Caruso, S	2022	October 2017 to November 2019	aged 18 to 39 years	SY, IM	CHCs / progestins	197	
Carvalho, N.	2018	June 2016 to August 2017	aged 18 to 45 years	OP, IM	progestins / LNG-IUDs	103	
Cheewadhanaraks, S.	2012	September 2007 to October 2011	aged 18 to 40 years	SY	progestin/OAC	84	
D'Hooghe, T.	2019	December 2012 to July 2015	aged 18 to 45 years	SY, OP	GnRH antagonists / placebo	540	
Diamond, M.P.	2014	2008-2009	aged 18 to 50 years	OP, SY	GnRH antagonists / placebo	155	
Donnez, J.	2020	2016-2017	aged 18 to 45 years	OP, SY	GnRH antagonists / placebo	328	
El Taha, E.	2021	February 2017 to October 2020	aged 20 to 45 years	OP, SY, IM	progestins / CHCs	70	
Ferreira, R.A.	2010	na	aged 18 to 40 years	OP	LNG-IUDs / GnRH agonists	44	
Giudice, L.C.	2022	December 2017 to December 2019	aged 18 to 50 years	OP, SY, IM	GnRH combination / GnRH antagonists/ placebo	638	SPIRIT 1
Giudice, L.C.	2022	November 2017 to October 2019	aged 18 to 50 years	OP, SY, IM	GnRH combination / GnRH antagonists/ placebo	623	SPIRIT 2
Guzick, D.S.	2011	2005–2008	aged 18 to premenopausal	OP, SY	CHCs / GnRH agonists + progestins	47	
Harada, T.	2008	na	aged over 18 years	OP, SY,IM	CHCs / placebo	100	
Harada, T.	2009	2003-2005	aged over 20 years	OP, IM, SY	progestins / GnRH agonists	271	
Harada, T.	2022	May 2019 to June 2020	aged over 20 years	OP, SY, IM	GnRH antagonists/ GnRH analog	454	
Harada, T.	2017	October 2012 to December 2014	aged over 20 years	OP, SY	progestins / CHCs / placebo	312	
Harrison, R.F.	2000	na	aged 20 to 39 years	OP	progestins / placebo	100	
Kashi, A.M.	2022	March 2018 to March 2020	aged 18 to 45 years	OP,SY	progestins / CHCs	108	
Lang, J.	2018	March 2013 to April 2015	aged 18 to 45 years	OP,SY	progestins / placebo	262	

Margatho, D.	2020	June 2016 to January 2019	aged 18 to 45 years	OP, SY, IM	progestins / LNG-IUDs	52	
Margatho, D.	2018	June 2016 to October 2017	aged 18 to 45 years	OP, SY, IM	progestins / LNG-IUDs	103	
Niakan, G.	2021	March 2018 to March 2020	aged 18 to 45 years	OP	progestins / placebo	108	
No authors listed	1996	na	aged 18 to 40 years	OP	progestins / GnRH agonists / placebo	55	
Osuga, Y.	2021	December 2011 to September 2013	aged 20 to 50 years	OP, SY, IM	GnRH antagonists / GnRH agonists / placebo	487	
Osuga, Y.	2021	March 2012 to February 2014	aged 20 to 50 years	OP, SY, IM	GnRH antagonists / GnRH agonists / placebo	397	
Stratton, P.	2008	January 1999 to December 2004	aged 18 to 45 years	OP, SY	GnRH antagonists / placebo	158	
Strowitzki, T.	2010	na	aged 18 to 45 years	OP	progestins / placebo	198	
Strowitzki, T.	2010	1998-2001	aged 18 to 45 years	OP	progestins / GnRH	252	
Strowitzki, T.	2012	na	aged 18 to 45 years		progestin/GnRH analog	252	
Takaesu, Y.	2016	April 2009 to June 2013	aged 18 years to before menopause	OP	progestins / GnRH agonists	111	
Tanmahasamut, P.	2017	April 2012 to October 2014	reproductive aged	OP,SY	progestins / placebo	40	
Taylor, H.S.	2017	2012-2014	aged 18 to 49 years	OP, SY	GnRH antagonists / placebo	872	Elaris EM1
Taylor, H.S.	2017	2013-2015	aged 18 to 49 years	OP, SY	GnRH antagonists / placebo	817	Elaris EM 2
Telimaa, S.	1987	na	premenopausal	OP	progestins / placebo	59	
Vercellini, P.	2005	na	aged 18 to 35 years	OP	CHCs / progestins	90	
Vercellini, P.	1994	na	aged 18 to 40 years	OP	danazol / GnRH agonists	42	
Vercellini, P.	1993	na	aged 18 to 35 years	OP, SY	GnRH agonists / CHCs	57	
Vercellini, P.	1996	na	aged 18 to 40 years		progestins / CHCs + danazol	80	
Vercellini, P.	2002	na	aged 18 to 40 years	OP, SY	progestins / CHCs	137	
Zhao, Y.	2021	June 2016 to June 2019	reproductive age	OP, SY	aromatase inhibitors + CHCs / CHCs	820	
Zupi, E.	2004	March 2000 to February 2003	aged 20 to 43 years	OP, SY	GnRH agonists + CHCs / CHCs	133	

Table S3. Final active ingredient groups following merging of substances.

1.	GnRH antagonists
2.	GnRH agonists
3.	CHCs
4.	progestins
5.	GnRH agonists + progestins
6.	aromatase inhibitors + progestins
7.	GnRH agonists + CHCs
8.	LNG-IUDs
9.	aromatase inhibitors + CHCs
10.	danazol
11.	CHCs + danazol
12.	GnRH agonists + aromatase inhibitors
13.	gestrinone
14.	mifepristone + gestrinone
15.	GnRH antagonist combination
16.	SERM

Table S4. Risk of bias assessment. D1: Randomization process, D1: Deviations from the intended interventions, D3: Missing outcome data, D4: Measurement of the outcome, D5: Selection of the reported result. PP: overall pelvic pain, DP: dyspareunia, DM: dysmenorrhea, 0–100: assessment on scale 0–100, 0–3: assessment on scale 0–3, 3M: 3-months follow-up time, 6M: 6-months follow-up time.

Study ID	Experimental	Comparator	Outcome	Weight	D1	D2	D3	D4	D5	Overall
Taylor, 2017	GnRH antagonists	placebo	PP 0-100 3M	1	+	+	+	+	+	+
Diamond, 2014	GnRH antagonists	placebo	PP 0-100 3M	1	+	+	+	+	+	+
Donnez, 2020	GnRH antagonists	placebo	PP 0-100 3M	1	+	+	+	+	+	+
D'Hooghe, 2019	GnRH antagonists	placebo	PP 0-100 3M	1	+	+	+	+	+	+
Bergqvist, 1998	GnRH agonists	placebo	PP 0-100 3M	1	+	+	+	+	+	+
Caruso, 2022	CHCs	progestins	PP 0-100 3M	1	+	+	+	-	+	-
Guzick, 2011	CHCs	GnRH agonists + progestins	PP 0-100 3M	1	+	+	+	+	+	+
Cheewadhanaraks, 2012	progestins	CHCs	PP 0-100 3M	1	+	+	+	-	+	-
Osuga, 2021	GnRH antagonists	placebo	PP 0-100 3M	1	+	+	+	+	+	+
Osuga, 2021	GnRH agonists	placebo	PP 0-100 3M	1	+	+	+	+	+	+
Osuga, 2021	GnRH agonists	GnRH antagonists	PP 0-100 3M	1	+	+	+	+	+	+
Harada, 2008	CHCs	placebo	PP 0-100 3M	1	+	+	+	+	+	+
El Taha, 2021	progestins	CHCs	PP 0-100 3M	1	+	+	+	+	+	+

Strowitzki, 2010	progestins	placebo	PP 0-100 3M	1	+	+	+	+	+	+
Lang, 2017	progestins	placebo	PP 0-100 3M	1	+	+	+	+	+	+
Abdou, 2018	progestins	GnRH agonists	PP 0-100 3M	1	+	+	+	-	+	-
Walch, 2008	progestins	progestins	PP 0-100 3M	1	+	+	+	-	+	-
Tekin, 2011	LNG-IUDs	GnRH agonists	PP 0-100 3M	1	+	+	+	-	+	-
Osuga, 2021/2	GnRH antagonists	placebo	PP 0-100 3M	1	+	+	+	+	+	+
Osuga, 2021/2	GnRH agonists	placebo	PP 0-100 3M	1	+	+	+	+	+	+
Harada, 2009	progestins	GnRH agonists	PP 0-100 6M	1	+	+	+	+	+	+
Bergqvist, 1998	GnRH agonists	placebo	PP 0-100 6M	1	+	+	+	+	+	+
Ferreira, 2010	LNG-IUDs	GnRH agonists	PP 0-100 6M	1	+	+	+	-	+	-
Ashraf, 2022	progestins + aromatase inhibitors	progestins	PP 0-100 6M	1	!	!	+	-	+	-
Margatho, 2018	progestins	LNG-IUDs	PP 0-100 6M	1	+	+	+	-	+	-
Caruso, 2022	CHCs	progestins	PP 0-100 6M	1	+	+	+	-	+	-
Carvalho, 2018	progestins	LNG-IUDs	PP 0-100 6M	1	+	+	+	-	+	-
Kashi, 2021	progestins	placebo	PP 0-100 6M	1	+	+	+	+	+	+
Kashi, 2021	CHCs	placebo	PP 0-100 6M	1	+	+	+	+	+	+
Kashi, 2021	CHCs	progestins	PP 0-100 6M	1	+	+	+	+	+	+

Zupi, 2004	GnRH agonists	CHCs	PP 0-100 6M	1	+	+	+	+	+	+
Takaesu, 2016	progestins	GnRH agonists	PP 0-100 6M	1	+	+	+	-	+	-
Zhao, 2020	aromatase inhibitors + CHCs	CHCs	PP 0-100 6M	1	+	+	+	+	+	+
Guzick, 2011	CHCs	GnRH agonists + progestins	PP 0-100 6M	1	+	+	+	+	+	+
Xue, 2016	mifepristone + gestrinone	gestrinone	PP 0-100 6M	1	+	+	!	-	+	-
Strowitzki, 2010/2	progestins	GnRH agonists	PP 0-100 6M	1	+	+	+	-	+	-
Harada, 2021	GnRH antagonists	GnRH agonists	PP 0-100 6M	1	+	+	+	+	+	+
Harada, 2017	progestins	placebo	PP 0-100 6M	1	+	+	+	+	+	+
Harada, 2017	CHCs	placebo	PP 0-100 6M	1	+	+	+	+	+	+
Harada, 2017	CHCs	progestins	PP 0-100 6M	1	+	+	+	+	+	+
El Taha, 2021	progestins	CHCs	PP 0-100 6M	1	+	+	+	+	+	+
Strowitzki, 2012	progestins	GnRH agonists	PP 0-100 6M	1	+	+	+	-	+	-
Lang, 2017	progestins	placebo	PP 0-100 6M	1	+	+	+	+	+	+
Walch, 2008	progestins	progestins	PP 0-100 6M	1	+	+	+	-	+	-
Tekin, 2011	LNG-IUDs	GnRH agonists	PP 0-100 6M	1	+	+	+	-	+	-
Haarrison, 2000	progestins	placebo	PP 0-3 3M	1	+	+	+	+	+	+
Ács, 2015	GnRH antagonists	placebo	PP 0-3 3M	1	+	+	+	+	+	+

Ács, 2015	GnRH agonists	placebo	PP 0-3 3M	1	+	+	+	+	+	+
Ács, 2015	GnRH agonists	GnRH antagonists	PP 0-3 3M	1	+	+	+	+	+	+
Telimaa, 1987	Danazol	placebo	PP 0-3 3M	1	!	+	+	+	+	!
Telimaa, 1987	progestins	placebo	PP 0-3 3M	1	!	+	+	+	+	!
Telimaa, 1987	progestins	Danazol	PP 0-3 3M	1	!	+	+	+	+	!
Abdou, 2018	progestins	GnRH agonists	DP 0-100 3M	1	+	+	+	-	+	-
Niakan, 2021	progestins	placebo	DP 0-100 3M	1	+	+	+	+	+	+
Niakan, 2021	CHCs	placebo	DP 0-100 3M	1	+	+	+	+	+	+
Niakan, 2021	CHCs	progestins	DP 0-100 3M	1	+	+	+	+	+	+
Stratton, 2008	SERMs	placebo	DP 0-100 3M	1	+	+	+	+	+	+
Vercellini, 2005	CHCs	progestins	DP 0-100 3M	1	+	+	+	-	+	-
Osuga, 2021	GnRH antagonists	placebo	DP 0-100 3M	1	+	+	+	+	+	+
Osuga, 2021	GnRH agonists	placebo	DP 0-100 3M	1	+	+	+	+	+	+
Osuga, 2021	GnRH agonists	GnRH antagonists	DP 0-100 3M	1	+	+	+	+	+	+
Kashi, 2021	progestins	placebo	DP 0-100 6M	1	+	+	+	+	+	+
Kashi, 2021	CHCs	placebo	DP 0-100 6M	1	+	+	+	+	+	+
Zupi, 2004	GnRH agonists	CHCs	DP 0-100 6M	1	+	+	+	+	+	+

Zhao, 2020	aromatase inhibitors + CHCs	CHCs	DP 0-100 6M	1	+	+	+	+	+	+
Vercellini, 1993	GnRH agonists	CHCs	DP 0-100 6M	1	+	+	+	-	+	-
Stratton, 2008	SERMs	placebo	DP 0-100 6M	1	+	+	+	+	+	+
Vercellini, 2005	CHCs	progestins	DP 0-100 6M	1	+	+	+	-	+	-
Xue, 2016	mifepristone + gestrinone	gestrinone	DP 0-100 6M	1	+	+	!	-	+	-
TGISG, 1996	gestrinone	GnRH agonists	DM 0-3 3M	1	+	+	+	+	+	+
D'Hooghe, 2019	GnRH antagonists	placebo	DM 0-3 3M	1	+	+	+	+	+	+
Ács, 2015	GnRH antagonists	placebo	DM 0-3 3M	1	+	+	+	+	+	+
Ács, 2015	GnRH agonists	placebo	DM 0-3 3M	1	+	+	+	+	+	+
Ács, 2015	GnRH agonists	GnRH antagonists	DM 0-3 3M	1	+	+	+	+	+	+
Stratton, 2008	SERMs	placebo	DM 0-100 3M	1	+	+	+	+	+	+
Vercellini, 2005	CHCs	progestins	DM 0-100 3M	1	+	+	+	-	+	-
Carvalho, 2018	progestins	LNG-IUDs	DM 0-100 6M	1	+	+	+	-	+	-
Zupi, 2004	GnRH agonists	CHCs	DM 0-100 6M	1	+	+	+	+	+	+
Takaesu, 2016	progestins	GnRH agonists	DM 0-100 6M	1	+	+	+	-	+	-
Zhao, 2020	aromatase inhibitors + CHCs	CHCs	DM 0-100 6M	1	+	+	+	+	+	+
Vercellini, 2005	CHCs	progestins	DM 0-100 6M	1	+	+	+	-	+	-

Vercellini, 1993	GnRH agonists	OAC	DM 0-100 6M	1	+	+	+	-	+	-
Stratton, 2008	SERMs	placebo	DM 0-100 6M	1	+	+	+	+	+	+
Xue, 2016	mifepristone + gestrinone	gestrinone	DM 0-100 6M	1	+	+	!	-	+	-
Osuga, 2021	GnRH antagonists	placebo	DM 0-100 3M	1	+	+	+	+	+	+
Osuga, 2021	GnRH agonists	placebo	DM 0-100 3M	1	+	+	+	+	+	+
Osuga, 2021	GnRH agonists	GnRH antagonists	DM 0-100 3M	1	+	+	+	+	+	+
Tanmahasamut, 2017	progestins	placebo	PP 0-100 6M	1	+	+	+	+	+	+
Vercellini, 1994	CHCs + danazol	GnRH agonists	DP 0-100 3M	1	+	+	+	-	+	-
Tanmahasamut, 2017	progestins	placebo	DM 0-3 6M	1	+	+	+	+	+	+
Vercellini, 1994	CHCs + danazol	GnRH agonists	DM 0-100 3M	1	+	+	+	-	+	-
Margatho, 2020	progestins	LNG-IUDs	PP 0-100 6M	1	+	+	+	-	+	-
Margatho, 2020	progestins	LNG-IUDs	DM 0-100 6M	1	+	+	+	-	+	-
Vercellini, 1996	progestins	CHCs + danazol	DP 0-100 6M	1	+	+	+	-	+	-
Vercellini, 1996	progestins	CHCs + danazol	DM 0-100 6M	1	+	+	+	-	+	-
SPIRIT1, 2022	GnRH antagonists	placebo	DM 0-100 3M	1	+	+	+	+	+	+
SPIRIT1, 2022	GnRH antagonist combination	placebo	DM 0-100 3M	1	+	+	+	+	+	+
SPIRIT1, 2022	GnRH antagonist combination	GnRH antagonists	DM 0-100 3M	1	+	+	+	+	+	+

SPIRIT1, 2022	GnRH antagonist combination	placebo	DM 0-100 6M	1	+	+	+	+	+	+
SPIRIT2, 2022	GnRH antagonists	placebo	DM 0-100 3M	1	+	+	+	+	+	+
SPIRIT2, 2022	GnRH antagonist combination	placebo	DM 0-100 3M	1	+	+	+	+	+	+
SPIRIT2, 2022	GnRH antagonist combination	GnRH antagonists	DM 0-100 3M	1	+	+	+	+	+	+
SPIRIT2, 2022	GnRH antagonist combination	placebo	DM 0-100 6M	1	+	+	+	+	+	+
Vercellini, 2002	progestins	CHCs	DM 0-100 6M	1	+	+	+	-	+	-
Vercellini, 2002	progestins	CHCs	DP 0-100 6M	1	+	+	+	-	+	-

+ Low risk

! Some concerns

- High risk

Table S5. CINeMA grade assessment.

Comparison	Number of Studies	Within-study bias	Reporting bias	Indirectness	Imprecision	Heterogeneity	Incoherence	Confidence rating	Reason(s) for downgrading
				Mixed evidence					
CHCs vs GnRH agonists	5	No concerns	Some concerns	Some concerns	Some concerns	Some concerns	Major concerns	Very low	
CHCs vs aromatase inhibitors plus CHCs	3	No concerns	Some concerns	Some concerns	No concerns	No concerns	Major concerns	Very low	
CHCs vs placebo	7	No concerns	Some concerns	Some concerns	Some concerns	Some concerns	No concerns	Low	
CHCs vs progestins	8	No concerns	Some concerns	Some concerns	Some concerns	Some concerns	Major concerns	Very low	
GnRH agonists vs GnRH antagonists	5	No concerns	Some concerns	Some concerns	Some concerns	Some concerns	No concerns	Low	
GnRH agonists vs LNG-IUDs	3	Major concerns	Some concerns	Some concerns	Some concerns	Some concerns	Some concerns	Very low	
GnRH agonists vs aromatase inhibitors	1	No concerns	Some concerns	Some concerns	Major concerns	No concerns	Major concerns	Very low	
GnRH agonists vs placebo	6	No concerns	Some concerns	Some concerns	No concerns	Major concerns	No concerns	Very low	
GnRH agonists vs progestins	7	No concerns	Some concerns	Some concerns	No concerns	Some concerns	No concerns	Low	

GnRH antagonista vs placebo	3	No concerns	Some concerns	Some concerns	Some concerns	No concerns	Major concerns	Very low
GnRH antagonists vs placebo	4	No concerns	Some concerns	Some concerns	Some concerns	Some concerns	No concerns	Low
LNG-IUDs vs progestins	3	Major concerns	Some concerns	Some concerns	No concerns	Some concerns	Major concerns	Very low
SERM vs placebo	3	No concerns	Some concerns	Some concerns	No concerns	Some concerns	Major concerns	Very low
SERMs vs placebo	1	No concerns	Some concerns	Some concerns	Some concerns	No concerns	Major concerns	Very low
placcebo vs progestins	2	No concerns	Some concerns	Some concerns	Some concerns	No concerns	Major concerns	Very low
placebo vs progestins	6	No concerns	Some concerns	Some concerns	Some concerns	No concerns	No concerns	Low
progestins vs progestins plus aromatase inhibitos	1	Major concerns	Some concerns	Some concerns	Major concerns	No concerns	Major concerns	Very low
			Indirect evidence					
CHCs vs GnRH antagonista	--	No concerns	Some concerns	Some concerns	Some concerns	No concerns	Major concerns	Very low
CHCs vs GnRH antagonists	--	No concerns	Some	Some	Major	No concerns	Major	Very low

CHCs vs LNG-IUDs	--	Major concerns	Some concerns	Some concerns	Some concerns	No concerns	Major concerns	Very low
CHCs vs SERM	--	No concerns	Some concerns	Some concerns	Some concerns	No concerns	Major concerns	Very low
CHCs vs SERMs	--	No concerns	Some concerns	Some concerns	Some concerns	Some concerns	Major concerns	Very low
CHCs vs aromatase inhibitors	--	No concerns	Some concerns	Some concerns	Major concerns	No concerns	Major concerns	Very low
CHCs vs placebo	--	No concerns	Some concerns	Some concerns	Some concerns	No concerns	Major concerns	Very low
CHCs vs progestins plus aromatase inhibitos	--	Major concerns	Some concerns	Some concerns	Major concerns	No concerns	Major concerns	Very low
GnRH agonists vs GnRH antagonista	--	No concerns	Some concerns	Some concerns	Some concerns	Some concerns	Major concerns	Very low
GnRH agonists vs SERM	--	No concerns	Some concerns	Some concerns	No concerns	Some concerns	Major concerns	Very low
GnRH agonists vs SERMs	--	No concerns	Some concerns	Some concerns	Some concerns	No concerns	Major concerns	Very low
GnRH agonists vs aromatase inhibitors plus CHCs	--	No concerns	Some concerns	Some concerns	No concerns	Some concerns	Major concerns	Very low
GnRH agonists vs placebo	--	No concerns	Some concerns	Some concerns	No concerns	Some concerns	Major concerns	Very low

GnRH agonists vs progestins plus aromatase inhibitos	--	Major concerns	Some concerns	Some concerns	Major concerns	No concerns	Major concerns	Very low	|
GnRH antagonista vs GnRH antagonists	--	No concerns	Some concerns	Some concerns	Some concerns	No concerns	Major concerns	Very low	|
GnRH antagonista vs LNG-IUDs	--	No concerns	Some concerns	Some concerns	Major concerns	No concerns	Major concerns	Very low	|
GnRH antagonista vs SERM	--	No concerns	Some concerns	Some concerns	No concerns	No concerns	Major concerns	Very low	|
GnRH antagonista vs SERMs	--	No concerns	Some concerns	Some concerns	No concerns	Some concerns	Major concerns	Very low	|
GnRH antagonista vs aromatase inhibitors	--	No concerns	Some concerns	Some concerns	Major concerns	No concerns	Major concerns	Very low	|
GnRH antagonista vs aromatase inhibitors plus CHCs	--	No concerns	Some concerns	Some concerns	Major concerns	No concerns	Major concerns	Very low	|
GnRH antagonista vs placcebo	--	No concerns	Some concerns	Some concerns	No concerns	No concerns	Major concerns	Very low	|
GnRH antagonista vs progestins	--	No concerns	Some concerns	Some concerns	No concerns	Some concerns	Major concerns	Very low	|
GnRH antagonista vs progestins plus aromatase inhibitos	--	No concerns	Some concerns	Some concerns	Major concerns	No concerns	Major concerns	Very low	|
GnRH antagonists vs LNG-IUDs	--	No concerns	Some concerns	Some concerns	Some concerns	No concerns	Major concerns	Very low	|

GnRH antagonists vs SERM	--	No concerns	Some concerns	Some concerns	Some concerns	No concerns	Major concerns	Very low
GnRH antagonists vs SERMs	--	No concerns	Some concerns	Some concerns	Some concerns	Some concerns	Major concerns	Very low
GnRH antagonists vs aromatase inhibitors	--	No concerns	Some concerns	Some concerns	Major concerns	No concerns	Major concerns	Very low
GnRH antagonists vs aromatase inhibitors plus CHCs	--	No concerns	Some concerns	Some concerns	No concerns	Some concerns	Major concerns	Very low
GnRH antagonists vs placcebo	--	No concerns	Some concerns	Some concerns	Some concerns	No concerns	Major concerns	Very low
GnRH antagonists vs progestins	--	No concerns	Some concerns	Some concerns	Some concerns	Some concerns	Major concerns	Very low
GnRH antagonists vs progestins plus aromatase inhibitos	--	No concerns	Some concerns	Some concerns	Major concerns	No concerns	Major concerns	Very low
LNG-IUDs vs SERM	--	No concerns	Some concerns	Some concerns	No concerns	No concerns	Major concerns	Very low
LNG-IUDs vs SERMs	--	No concerns	Some concerns	Some concerns	No concerns	Some concerns	Major concerns	Very low
LNG-IUDs vs aromatase inhibitors	--	No concerns	Some concerns	Some concerns	Major concerns	No concerns	Major concerns	Very low
LNG-IUDs vs aromatase inhibitors plus CHCs	--	No concerns	Some concerns	Some concerns	Major concerns	No concerns	Major concerns	Very low

LNG-IUDs vs placcebo	--	No concerns	Some concerns	Some concerns	No concerns	No concerns	Major concerns	Very low
LNG-IUDs vs placebo	--	No concerns	Some concerns	Some concerns	Some concerns	No concerns	Major concerns	Very low
LNG-IUDs vs progestins plus aromatase inhibitos	--	Major concerns	Some concerns	Some concerns	Some concerns	Some concerns	Major concerns	Very low
SERM vs SERMs	--	No concerns	Some concerns	Some concerns	Major concerns	No concerns	Major concerns	Very low
SERM vs aromatase inhibitors	--	No concerns	Some concerns	Some concerns	Some concerns	No concerns	Major concerns	Very low
SERM vs aromatase inhibitors plus CHCs	--	No concerns	Some concerns	Some concerns	No concerns	No concerns	Major concerns	Very low
SERM vs placcebo	--	No concerns	Some concerns	Some concerns	Major concerns	No concerns	Major concerns	Very low
SERM vs progestins	--	No concerns	Some concerns	Some concerns	Some concerns	No concerns	Major concerns	Very low
SERM vs progestins plus aromatase inhibitos	--	No concerns	Some concerns	Some concerns	Major concerns	No concerns	Major concerns	Very low
SERMs vs aromatase inhibitors	--	No concerns	Some concerns	Some concerns	Some concerns	Some concerns	Major concerns	Very low
SERMs vs aromatase inhibitors plus CHCs	--	No concerns	Some concerns	Some concerns	No concerns	No concerns	Major concerns	Very low

SERMs vs placcebo	--	No concerns	Some concerns	Some concerns	Major concerns	No concerns	Major concerns	Very low
SERMs vs progestins	--	No concerns	Some concerns	Some concerns	Major concerns	No concerns	Major concerns	Very low
SERMs vs progestins plus aromatase inhibitos	--	No concerns	Some concerns	Some concerns	Major concerns	No concerns	Major concerns	Very low
aromatase inhibitors vs aromatase inhibitors plus CHCs	--	No concerns	Some concerns	Some concerns	Some concerns	No concerns	Major concerns	Very low
aromatase inhibitors vs placcebo	--	No concerns	Some concerns	Some concerns	Some concerns	No concerns	Major concerns	Very low
aromatase inhibitors vs placebo	--	No concerns	Some concerns	Some concerns	Major concerns	No concerns	Major concerns	Very low
aromatase inhibitors vs progestins	--	No concerns	Some concerns	Some concerns	Some concerns	Some concerns	Major concerns	Very low
aromatase inhibitors vs progestins plus aromatase inhibitos	--	No concerns	Some concerns	Some concerns	Major concerns	No concerns	Major concerns	Very low
aromatase inhibitors plus CHCs vs placcebo	--	No concerns	Some concerns	Some concerns	No concerns	No concerns	Major concerns	Very low
aromatase inhibitors plus CHCs vs placebo	--	No concerns	Some concerns	Some concerns	No concerns	Some concerns	Major concerns	Very low
aromatase inhibitors plus CHCs vs progestins	--	No concerns	Some concerns	Some concerns	No concerns	No concerns	Major concerns	Very low

aromatase inhibitors plus CHCs vs progestins plus aromatase inhibitos	--	No concerns	Some concerns	Some concerns	Some concerns	No concerns	Major concerns	Very low
placcebo vs placebo	--	No concerns	Some concerns	Some concerns	Some concerns	No concerns	Major concerns	Very low
placcebo vs progestins plus aromatase inhibitos	--	No concerns	Some concerns	Some concerns	Major concerns	No concerns	Major concerns	Very low
placebo vs progestins plus aromatase inhibitos	--	Major concerns	Some concerns	Some concerns	Major concerns	No concerns	Major concerns	Very low

Figure S1. Network figure of dysmenorrhea on a scale of 0–100 after 3 months.

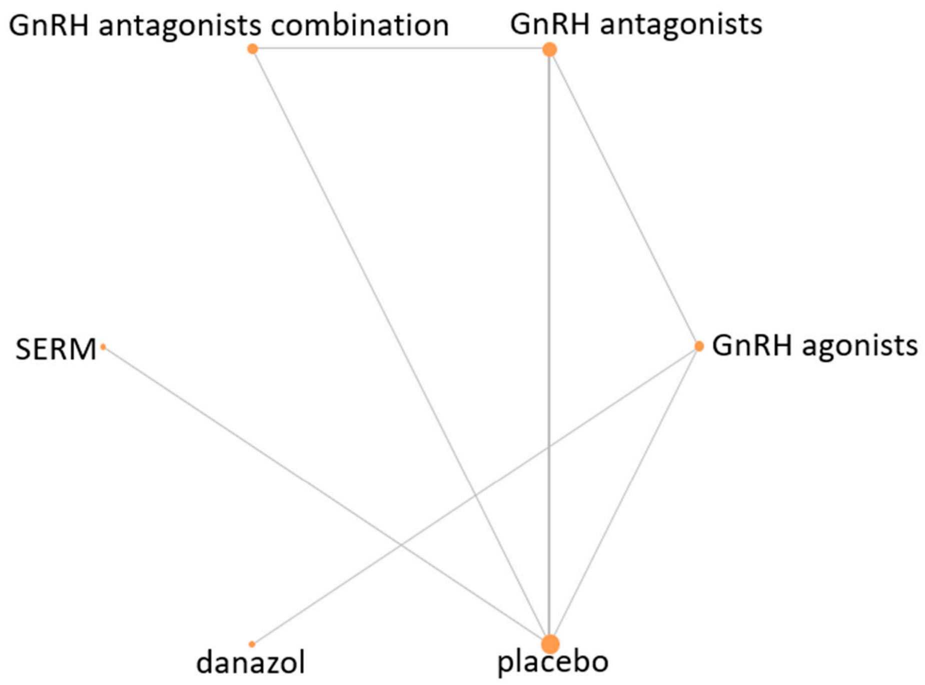

Figure S2. Ranking probability of dysmenorrhea on a scale of 0–100 after 3 months.

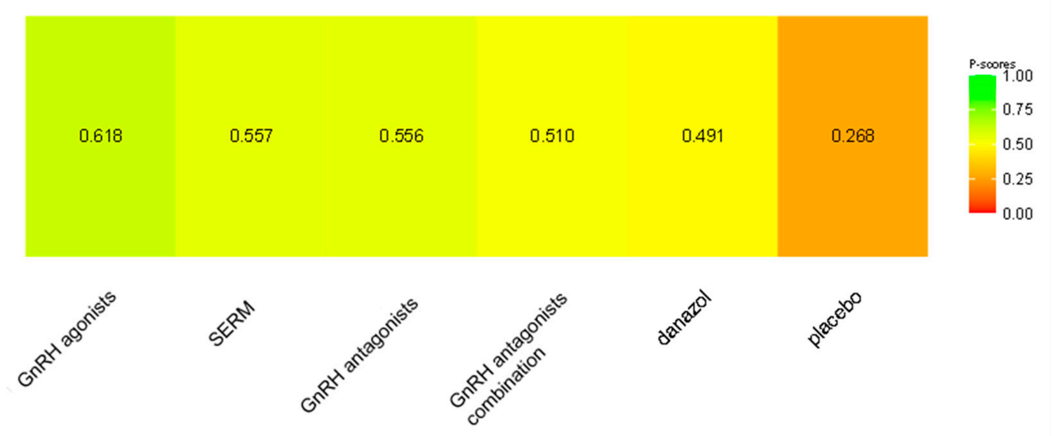

Figure S3. Forest plot of dysmenorrhea on a scale of 0–100 after 3 months.

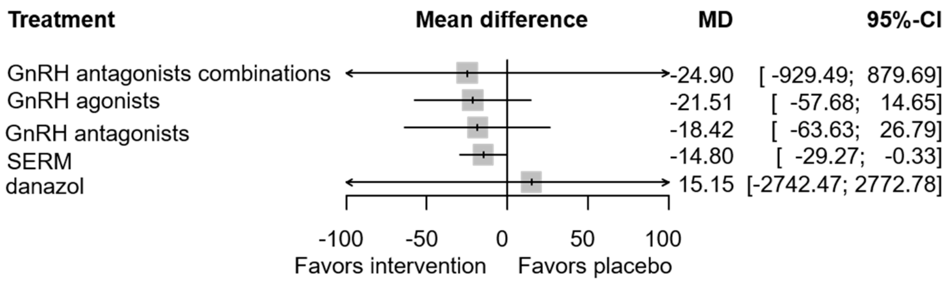

Figure S4. Sucra plot of dysmenorrhea on a scale of 0–100 after 3 months.

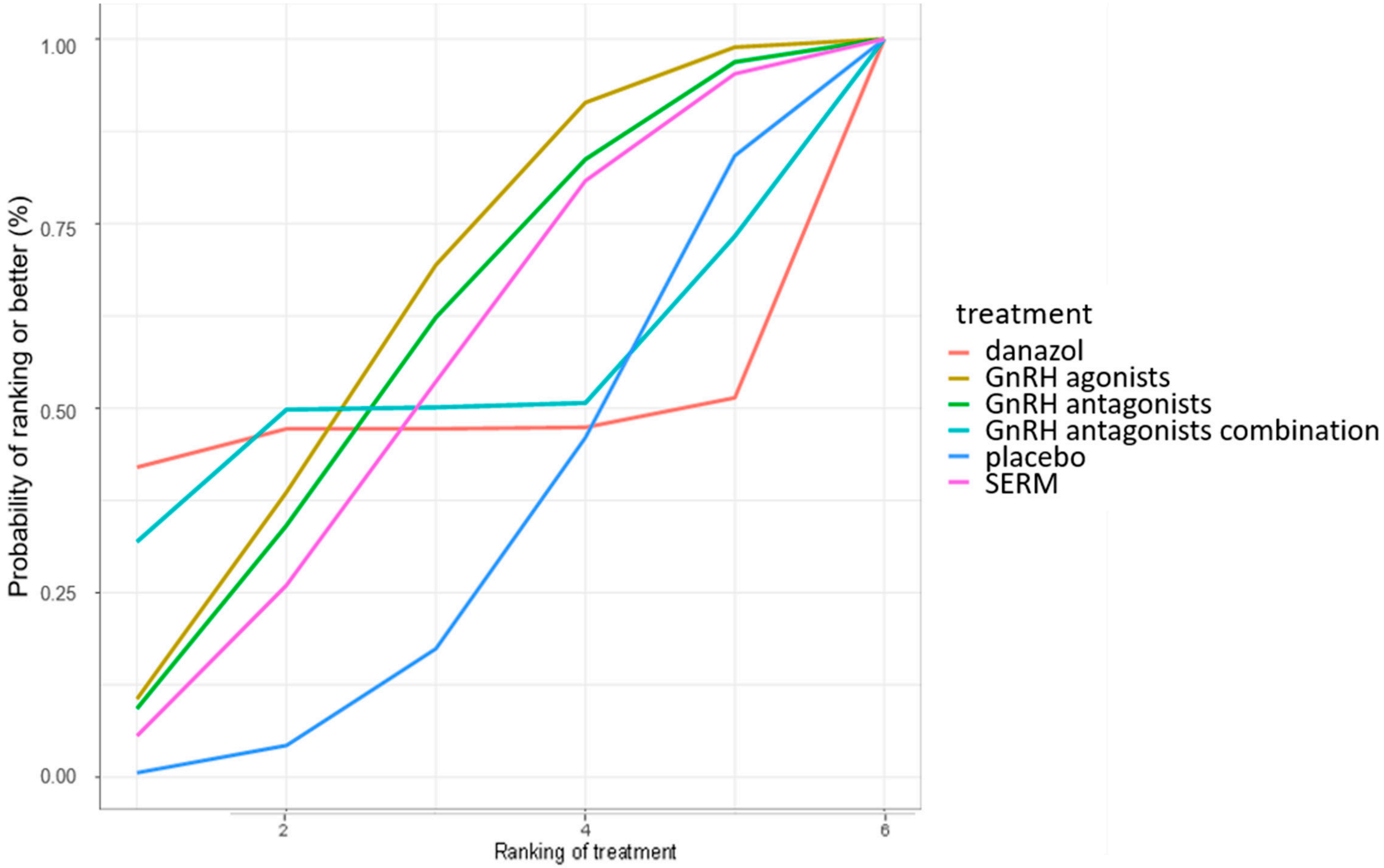

Figure S5. Network figure of dysmenorrhea on a scale of 0–3 after 3 months.

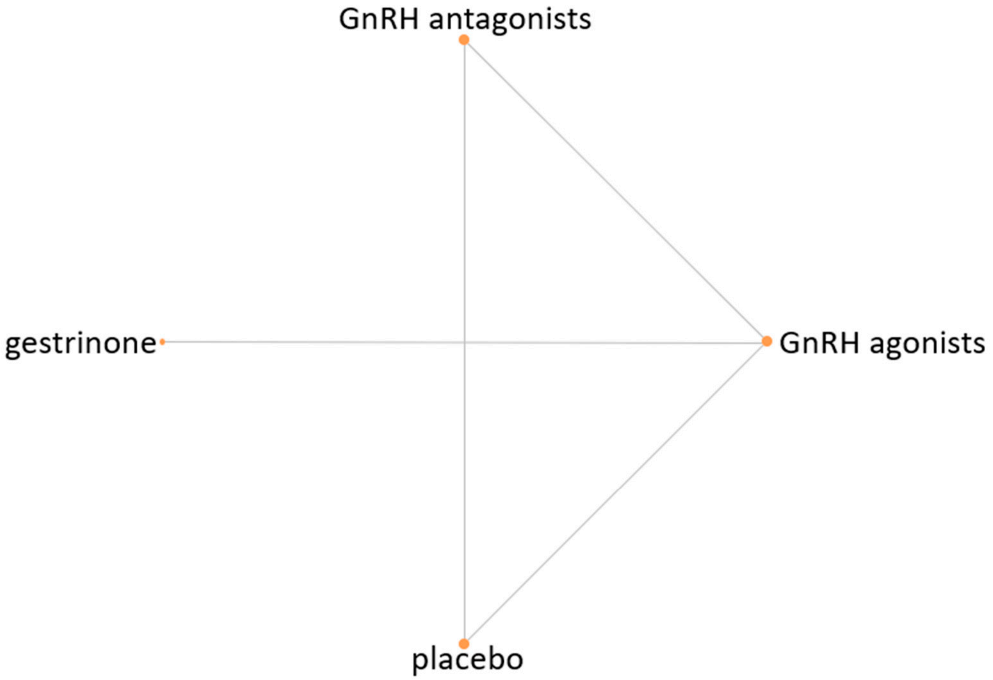

Figure S6. Ranking probability of dysmenorrhea on a scale of 0–3 after 3 months.

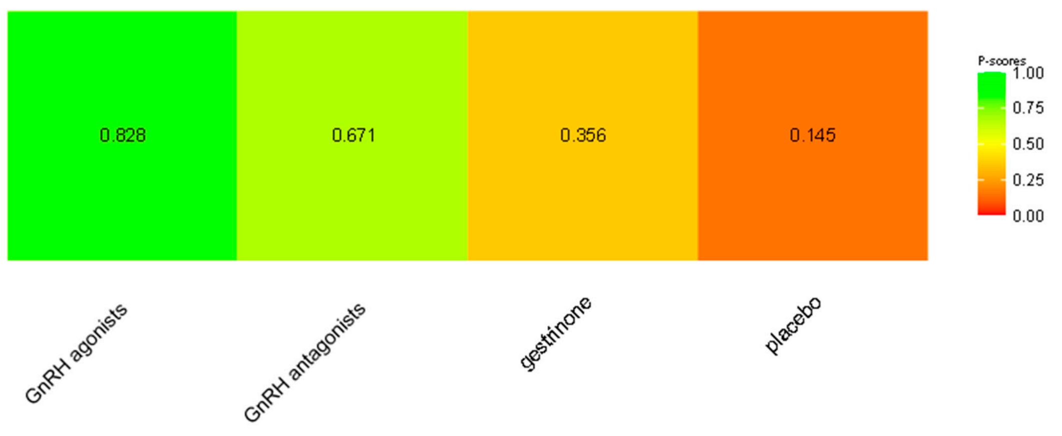

Figure S7. Forest plot of dysmenorrhea on a scale of 0–3 after 3 months.

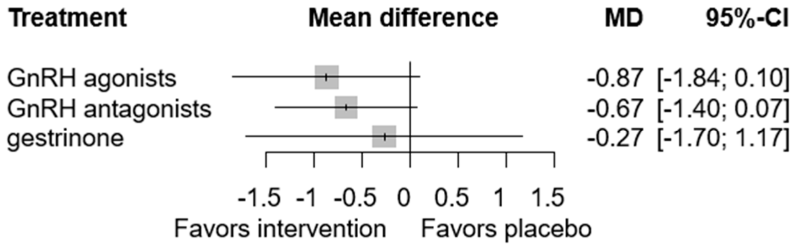

Figure S8. Sucra plot of dysmenorrhea on a scale of 0–3 after 3 months.

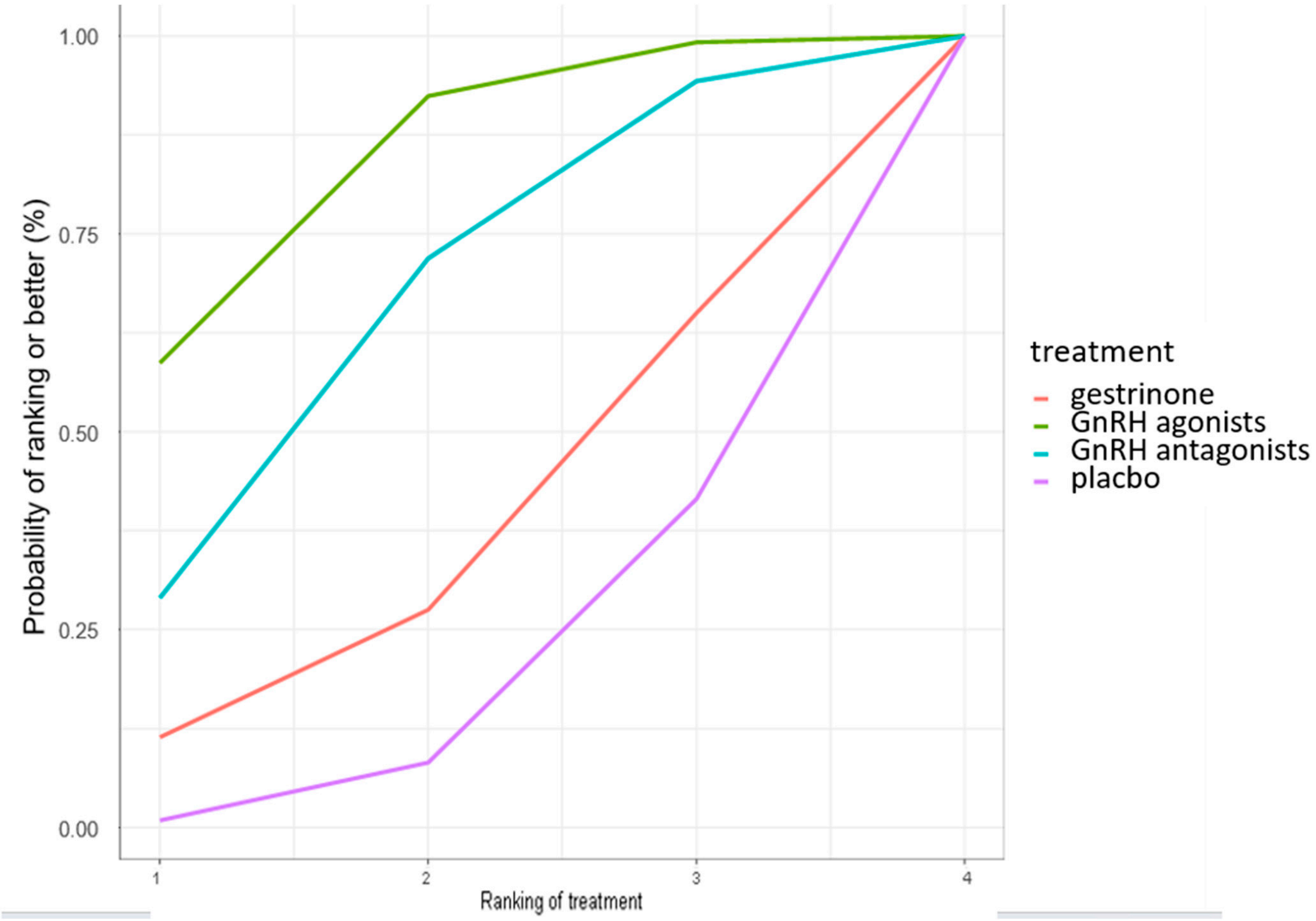

Figure S9. Network figure of dyspareunia on a scale of 0–100 after 3 months.

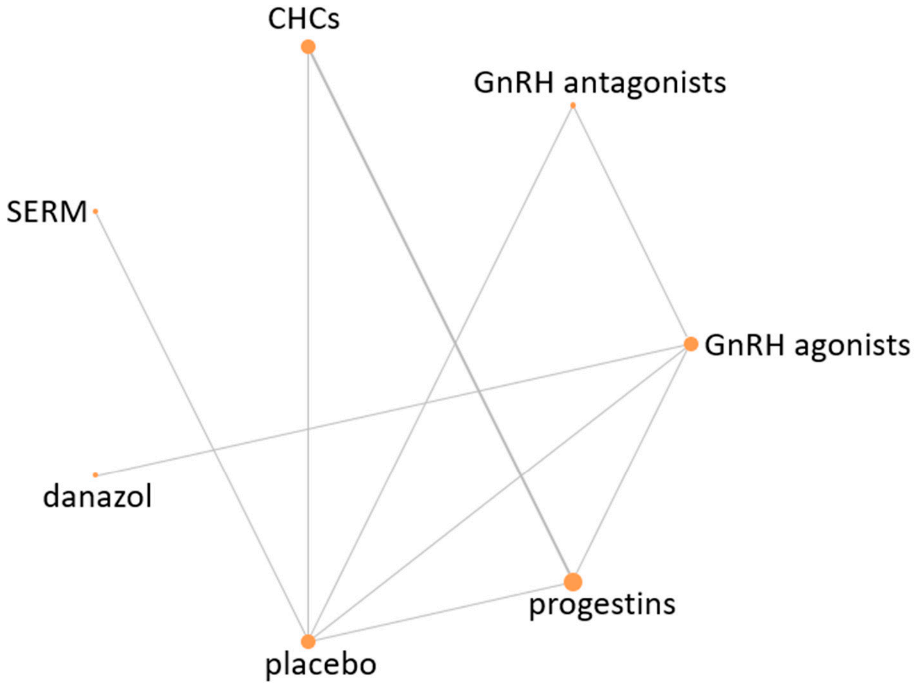

Figure S10. Ranking probability of dyspareunia on a scale of 0–100 after 3 months.

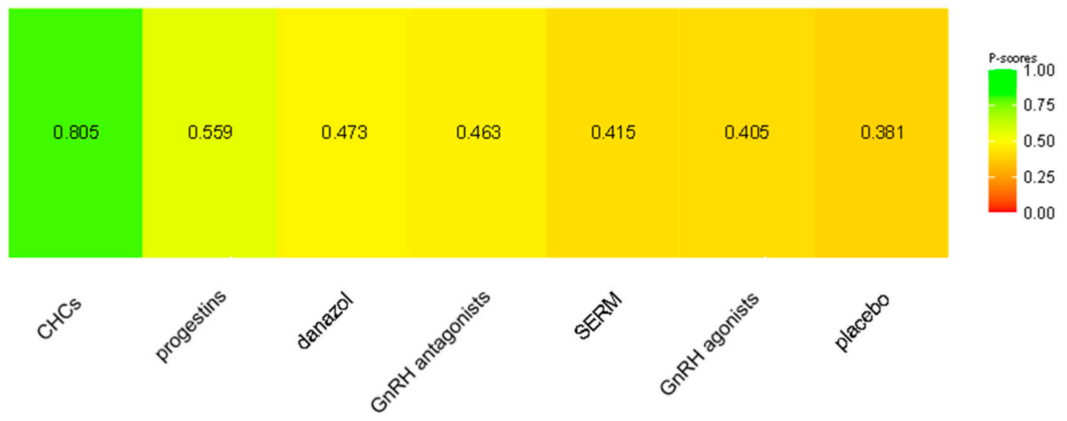

Figure S11. Forest plot of dyspareunia on a scale of 0–100 after 3 months.

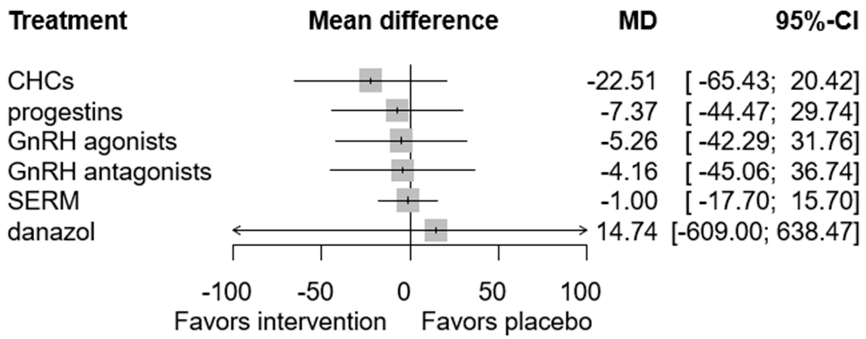

Figure S12. Sucra plot of dyspareunia on a scale of 0–100 after 3 months.

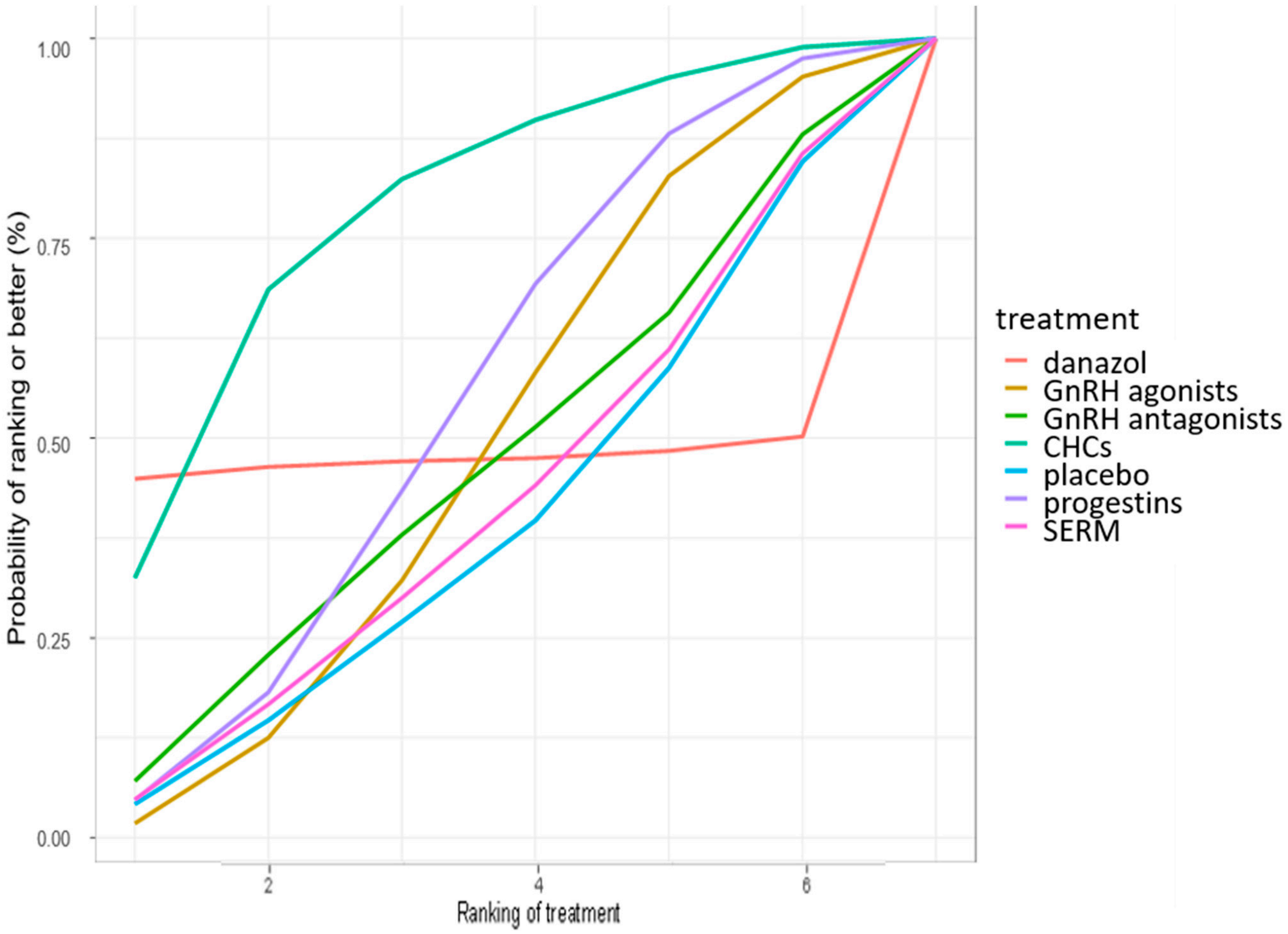

Figure S13. Network figure of dyspareunia on a scale of 0–100 after 6 months.

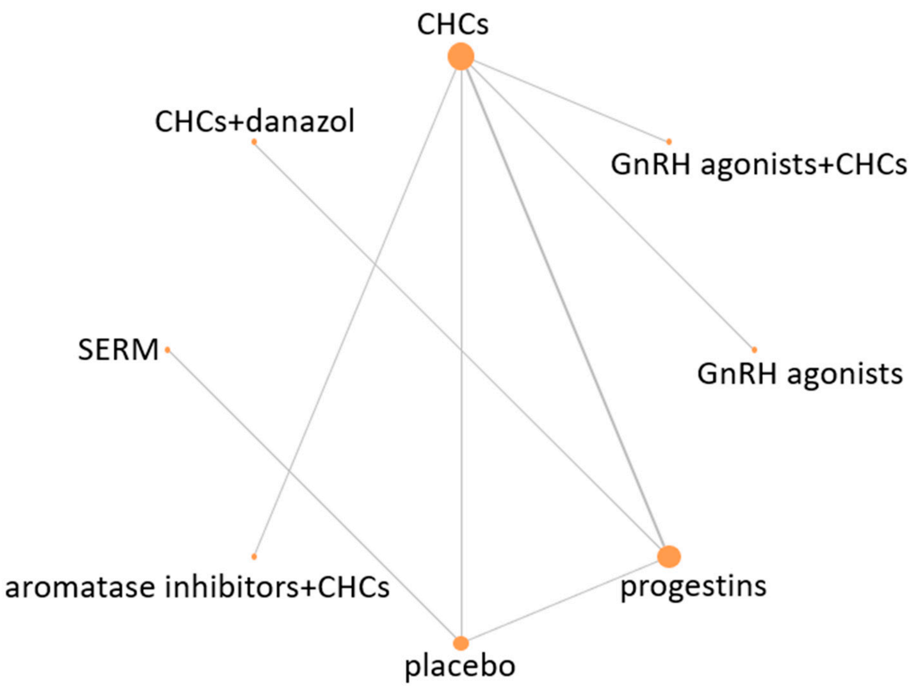

Figure S14. Ranking probability of dyspareunia on a scale of 0–100 after 6 months.

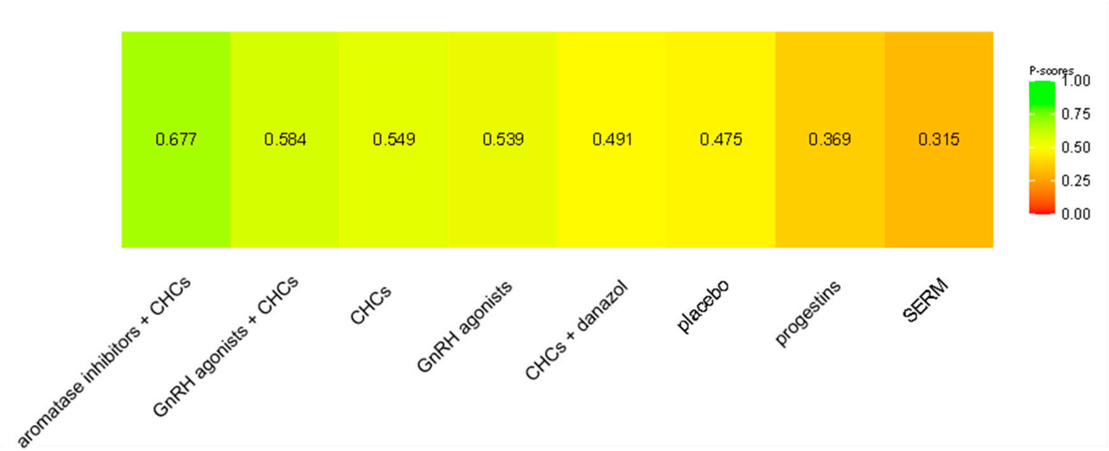

Figure S15. Forest plot of dyspareunia on a scale of 0–100 after 6 months.

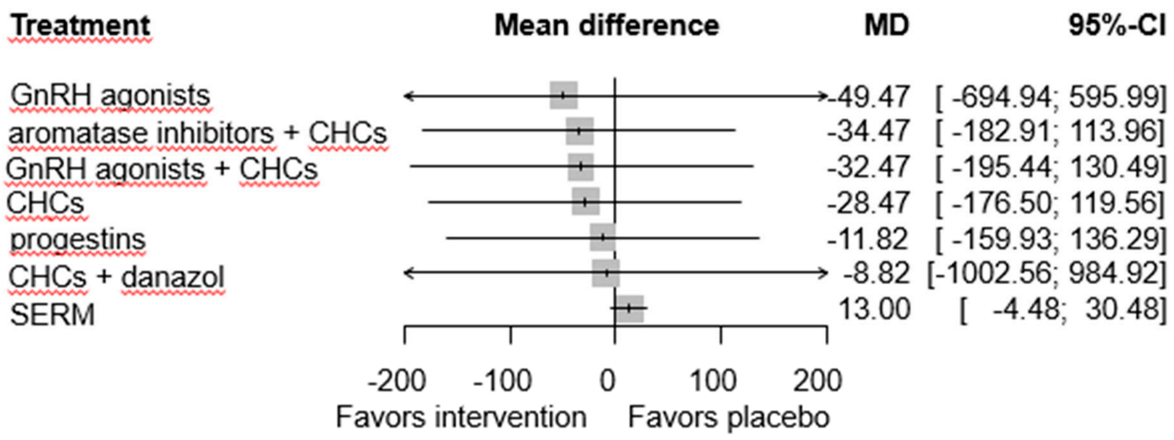

Figure S16. Sucra plot of dyspareunia on a scale of 0–100 after 6 months.

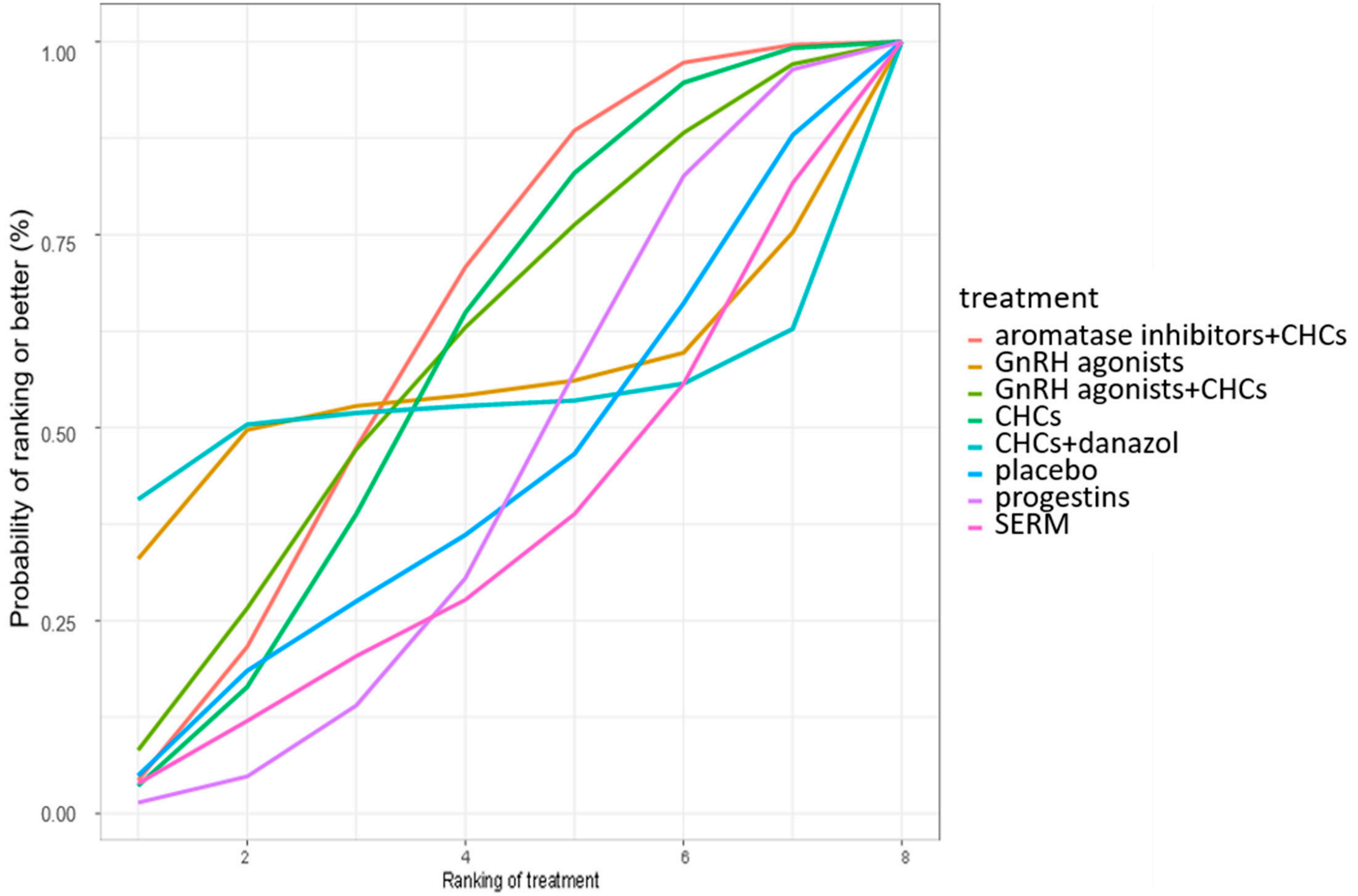

Figure S17. Network figure of overall pelvic pain on a scale of 0–100 after 3 months.

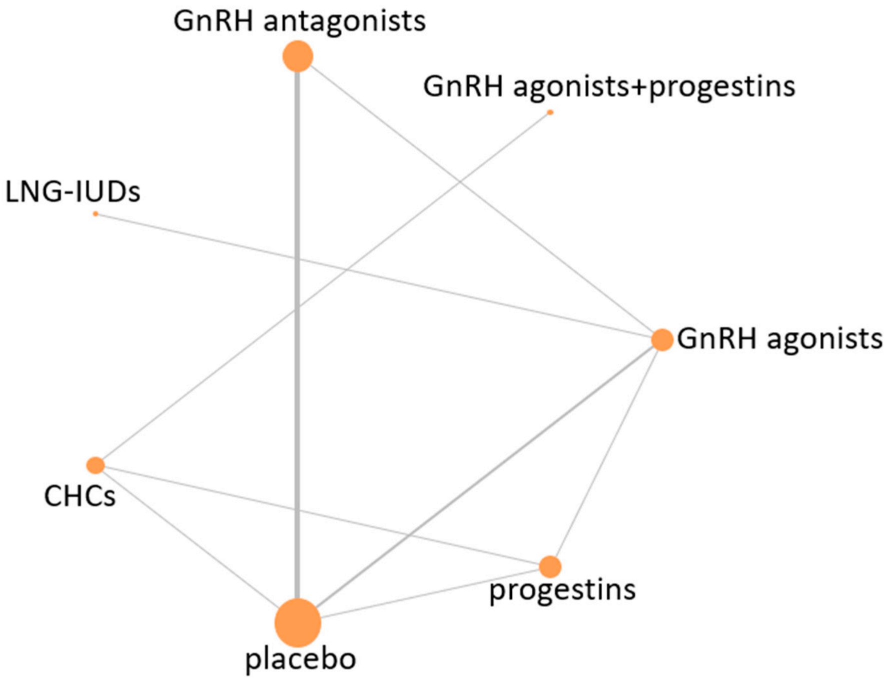

Figure S18. Ranking probability of overall pelvic pain on a scale of 0–100 after 3 months.

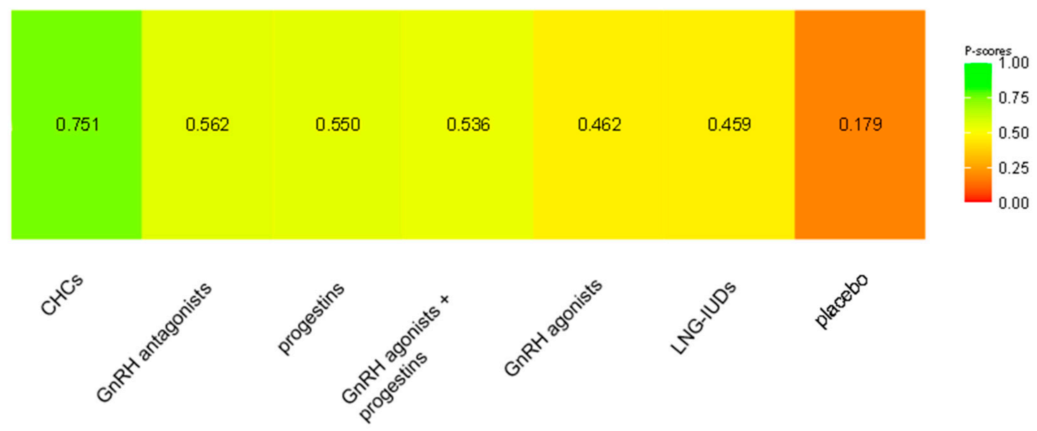

Figure S19. Forest plot of overall pelvic pain on a scale of 0–100 after 3 months.

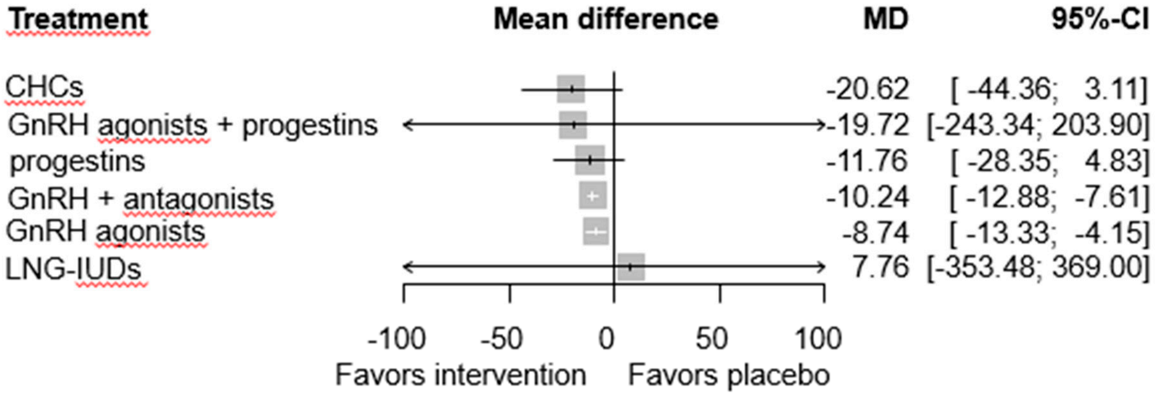

Figure S20. Sucra plot of overall pelvic pain on a scale of 0–100 after 3 months.

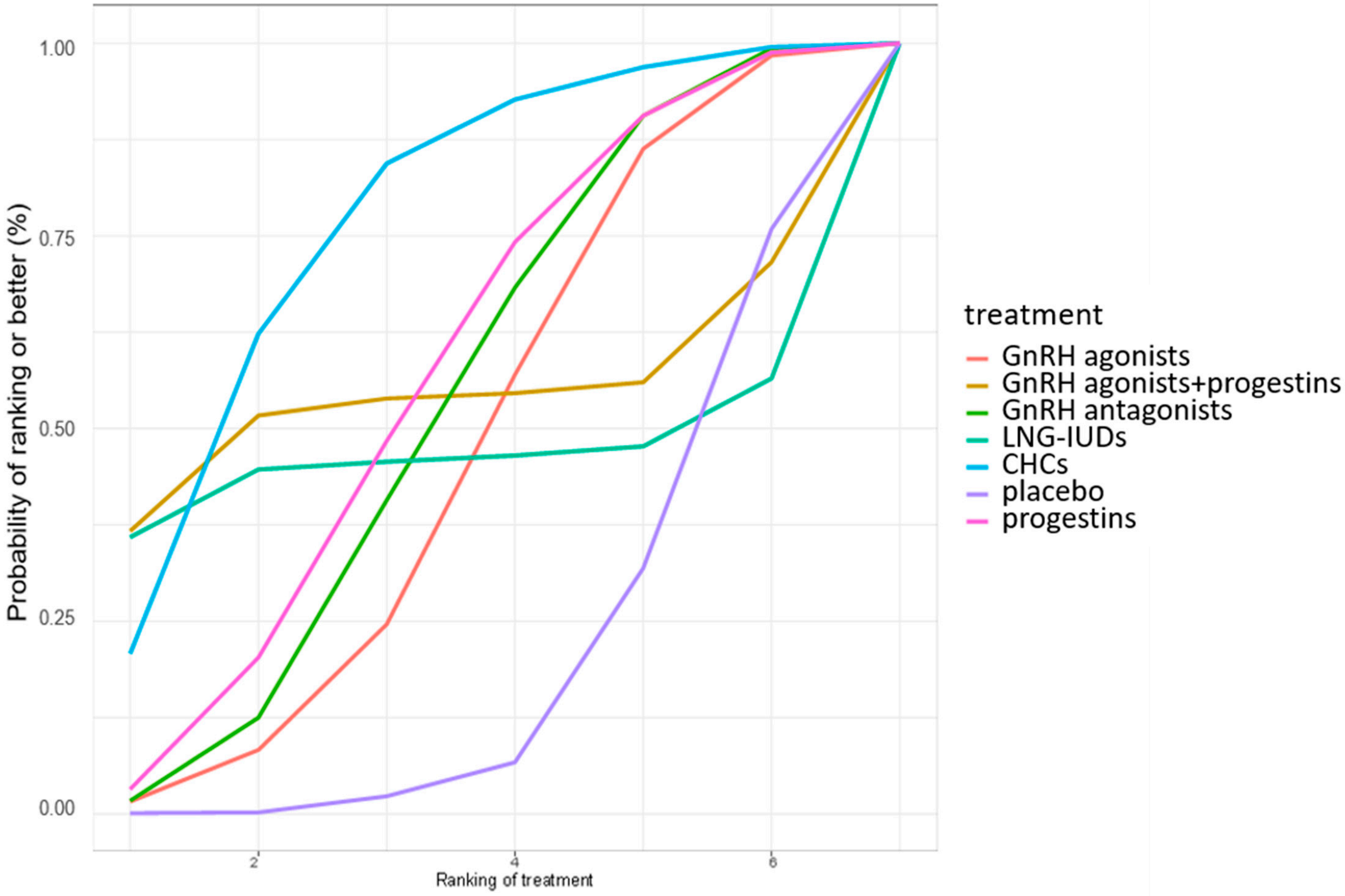

Figure S21. Network figure of overall pelvic pain on a scale of 0–3 after 3 months.

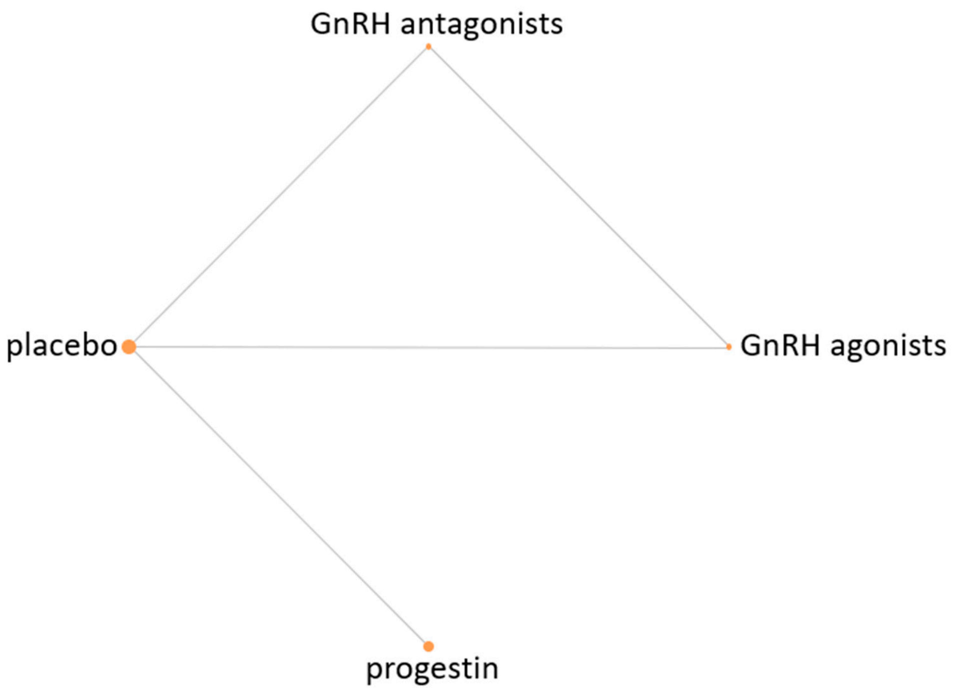

Figure S22. Ranking probability of overall pelvic pain on a scale of 0–3 after 3 months.

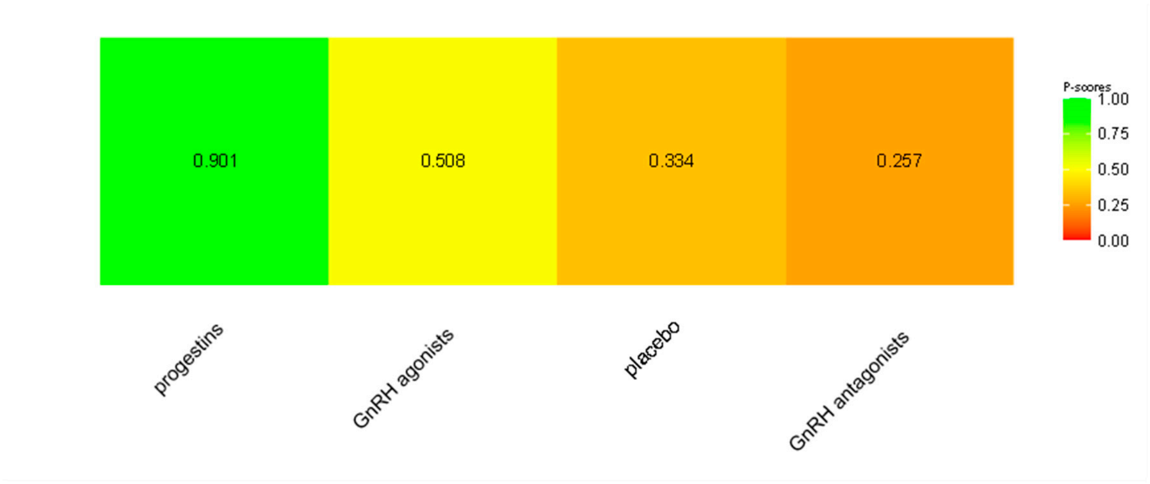

Figure S23. Forest plot of overall pelvic pain on a scale of 0–3 after 3 months.

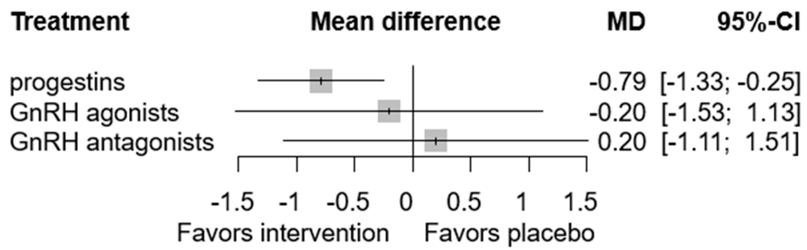

Figure S24. Sucra plot of overall pelvic pain on a scale of 0–3 after 3 months.

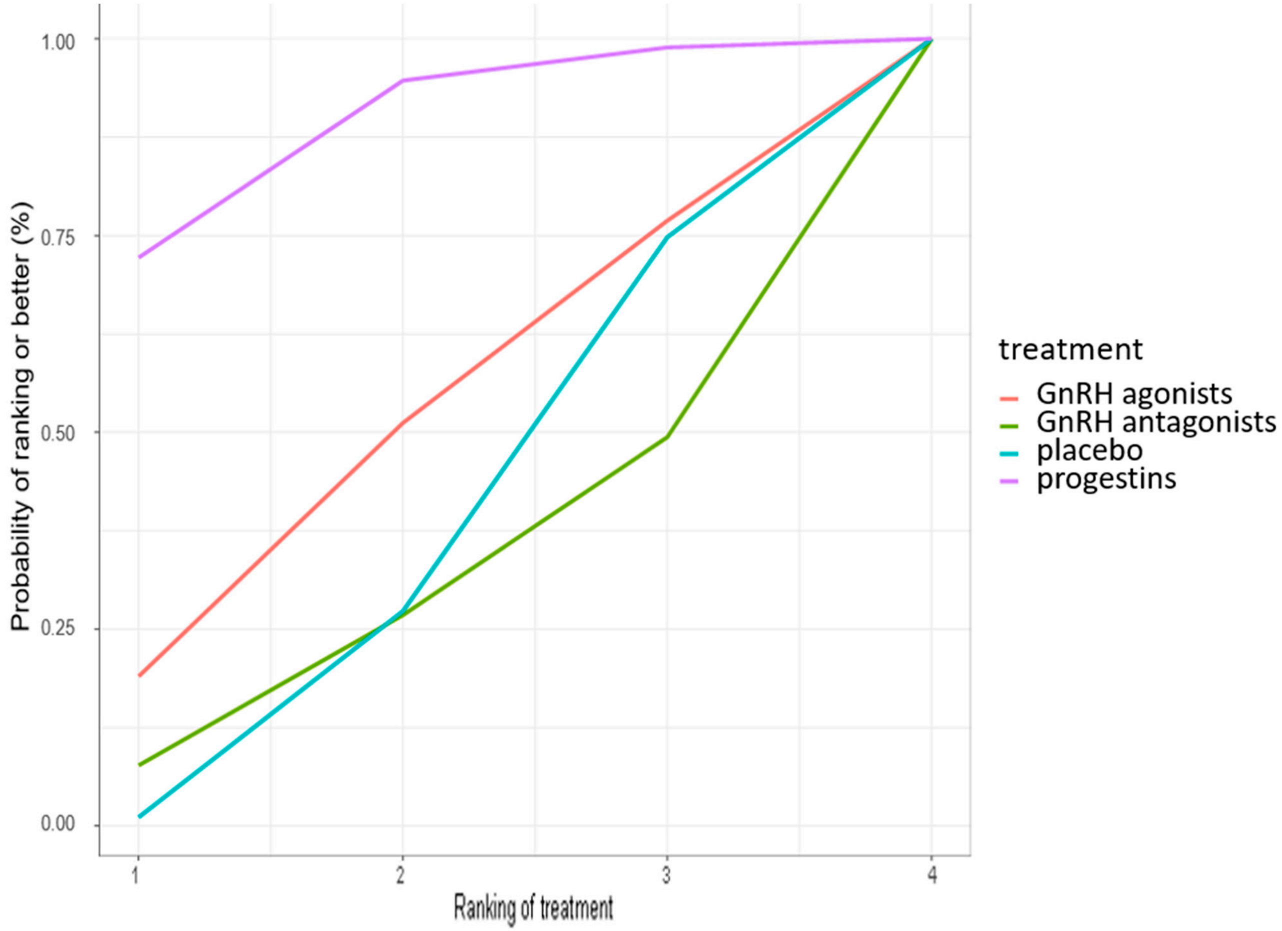

Supplementary S1. Search key

endometrio* **AND** (danazol OR gestrinone OR medroxyprogesterone OR opioid* OR norethisterone OR desogestrel OR cytoproterone OR combined hormonal contraceptive OR GnRH agonist OR linzagolix OR gonadotrophin-releasing hormone antagonist OR elagolix OR relugolix OR GnRH antagonist OR progestin OR mifepristone OR aromatase inhibitor OR "selective estrogen receptor modulator" OR SERM OR non-steroidal anti-inflammatory drugs OR NSAID) **AND** random* **AND** pain*.
